# Lipase regulation of cellular fatty acid homeostasis as a Parkinson’s disease therapeutic strategy

**DOI:** 10.1038/s41531-022-00335-6

**Published:** 2022-06-09

**Authors:** Saranna Fanning, Haley Cirka, Jennifer L. Thies, Jooyoung Jeong, Sarah M. Niemi, Joon Yoon, Gary P. H. Ho, Julian A. Pacheco, Ulf Dettmer, Lei Liu, Clary B. Clish, Kevin J. Hodgetts, John N. Hutchinson, Christina R. Muratore, Guy A. Caldwell, Kim A. Caldwell, Dennis Selkoe

**Affiliations:** 1 Ann Romney Center for Neurologic Diseases, Department of Neurology, Brigham and Women’s Hospital and Harvard Medical School, Boston, MA 02115 USA; 2 Department of Biological Sciences, The University of Alabama, Tuscaloosa, AL 35487 USA; 3 Department of Biostatistics, The Harvard Chan School of Public Health, Boston, MA 02115 USA; 4 Broad Institute of MIT and Harvard, Cambridge, MA 02142 USA; 5 Laboratory for Drug Discovery in Neuroscience, Department of Neurology, Brigham and Women’s Hospital, Boston, MA 02115 USA

**Keywords:** Cellular neuroscience, Parkinson's disease, Neurodegenerative diseases

## Abstract

Synucleinopathy (Parkinson’s disease (PD); Lewy body dementia) disease-modifying treatments represent a huge unmet medical need. Although the PD-causing protein α-synuclein (αS) interacts with lipids and fatty acids (FA) physiologically and pathologically, targeting FA homeostasis for therapeutics is in its infancy. We identified the PD-relevant target stearoyl-coA desaturase: inhibiting monounsaturated FA synthesis reversed PD phenotypes. However, lipid degradation also generates FA pools. Here, we identify the rate-limiting lipase enzyme, LIPE, as a candidate target. Decreasing LIPE in human neural cells reduced αS inclusions. Patient αS triplication vs. corrected neurons had increased pSer129 and insoluble αS and decreased αS tetramer:monomer ratios. LIPE inhibition rescued all these and the abnormal unfolded protein response. LIPE inhibitors decreased pSer129 and restored tetramer:monomer equilibrium in αS E46K-expressing human neurons. LIPE reduction in vivo alleviated αS-induced dopaminergic neurodegeneration in *Caenorhabditis elegans*. Co-regulating FA synthesis and degradation proved additive in rescuing PD phenotypes, signifying co-targeting as a therapeutic strategy.

## Introduction

There is a critical need to develop disease-modifying treatments for human synucleinopathies, most prominently Parkinson’s disease (PD) and Lewy body dementia (LBD). Lewy bodies (LBs) and Lewy neurites are the neuropathological hallmarks of PD, both in the sporadic (“idiopathic”) and autosomal dominant (familial) forms, and accumulate in Alzheimer’s disease (AD)^1–5^. α-Synuclein (αS) has been implicated since 1997 as the major proteinaceous component of LB^6,7^. Importantly, a recent publication also identified substantial lipid membrane components in LBs^8^.

Lipids contribute fundamentally to many cellular processes, including membrane synthesis, energy storage, signaling, and complex protein modifications. Membrane phospholipids are comprised of fatty acyl side chains that differ in carbon chain length and can be saturated or unsaturated, thereby largely determining membrane fluidity and influencing protein–protein and protein–lipid interactions. The brain is the second most lipid-rich organ in the body^9^. Lipid and FA homeostasis are essential determinants of neural development, neurotransmission, and receptor activation. Cells tightly regulate lipid synthesis, precursor uptake, and subcellular distribution, especially FAs. One ubiquitous homeostatic mechanism is the storage of FAs as triglycerides (TGs) in cytoplasmic lipid droplets (LDs) that help prevent cytotoxic consequences due to the accumulation of free FAs^10,11^. LDs are dynamic organelles present in most pro- and eukaryotic cells^12,13^. Notably, however, LDs are in relatively low abundance in neurons, suggesting that FA synthesis and metabolism maybe even more tightly regulated in the central nervous system to avoid detrimental excess^9,14^.

The disease-associated protein αS is a 14 kDa cytosolic polypeptide highly expressed in neurons. It has physiologic and pathogenic interactions with membrane phospholipids^15–18^ and with FAs^19–22^, and it can alter lipid homeostasis^23^. Overexpression of αS promotes LD formation^23–25^, and changes in LD content and distribution have been associated with αS toxicity, neurodegeneration, and membrane trafficking defects^26–28^, indicating that αS expression impacts FA homeostasis^23^. Mutations in several genes regulating lipids/FAs are associated with increased risk of PD, and multiple lipid species have been found to be altered in PD patient samples^29,30^. Indeed, a systematic GWAS analysis revealed lipids as a common factor among numerous PD-relevant processes, including oxidative stress response, endosomal-lysosomal functioning, ER stress response, and neuronal death^31^. Collectively, these data strongly suggest an αS/lipid interplay and thereby a potential role for lipids/FA in modulating PD/LBD-relevant αS phenotypes in the brain.

Emerging knowledge of lipid alterations in PD/LBD has recently identified a novel FA-related target, stearoyl-CoA desaturase (SCD), inhibition of which reverses numerous PD-relevant phenotypes in cells^23,32–34^ and in a PD mouse model^35^. SCD inhibitors have now reached human clinical trials for PD treatment. Having initially focused on the FA synthetic pathway for synucleinopathy, we have now systematically investigated another major source of cellular FAs, neutral lipid lipase, as a potential therapeutic target. This approach is functionally distinct from, but potentially just as important as, SCD inhibition. Given that excess or mutant αS expression results in increased monounsaturated FA production, it is likely that PD patients at the time of diagnosis already have LD accumulation in the brain^36–38^. Therefore, a potential concern is the continuous generation of more monounsaturated FAs through a degradation process.

Here, we identify LIPE, a triacylglycerol lipase, as a new PD-relevant therapeutic target. We find that LIPE regulates phospholipid-incorporated FA content (i.e., FAs in phospholipids), most prominently unsaturated FA levels^39,40^. This may be particularly important given that altering phospholipid membrane composition changes αS:membrane interactions^41^. We show that reducing LIPE activity decreased αS accumulation in round, membrane-rich cytoplasmic inclusions. Decreasing LIPE also reduced PD-associated phosphorylated αS and insoluble αS levels and decreased the unfolded protein response (UPR) in patient-derived αS triplication vs. isogenic corrected neurons. Importantly, LIPE inhibition increased the abnormally low αS tetramer:monomer ratio (T:M) in the αS triplication neurons as well as in human neurons expressing the familial PD (fPD) E46K αS mutation and increased the ratio of cytosolic to membrane-bound αS. Our genetic and pharmacological inhibition of LIPE strongly suggests that lowering unsaturated FA levels by slowing the lipase-mediated lipid degradation process represents a novel therapeutic strategy, a finding that stands in mechanistic agreement with our earlier work on SCD inhibition and augments the relevance of targeting FA metabolism to achieve αS homeostasis. Long-term SCD inhibitor treatment could result in secondary FA generation via LD/TG degradation to maintain FA levels. Therefore, we propose a treatment strategy incorporating partial inhibition of both synthesis and degradation by co-regulating SCD and LIPE. Indeed, we show that this approach is additive in reducing PD- and LBD-relevant phenotypes in neurons, including αS hyperphosphorylation.

## Results

### Genetic depletion of LIPE neutral lipid lipase activity decreases αS-positive inclusions

**Fig. 1:**
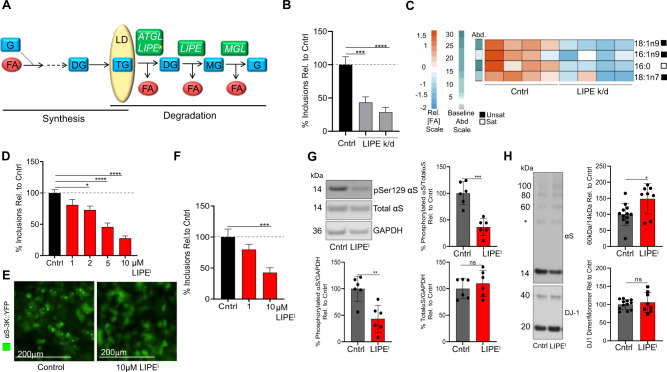
Reducing lipase activity genetically and pharmacologically reduces PD-relevant phenotypes in an αS-3K model. **A** Neutral lipid synthesis and degradation pathways. G glycerol, MG monoglyceride, DG diglyceride, TG triglyceride, FA fatty acid, *ATGL* adipose triglyceride lipase, *LIPE* hormone-sensitive lipase, *MGL* monoglyceride lipase, LD lipid droplet. * denotes rate-limiting step. Dotted line: upstream synthesis pathway. **B** LIPE knockdown decreases αS inclusions. M17D/αS-3K::YFP cells incubated (24 h) with shRNA targeting LIPE, αS expression induced for 24 h, number of inclusions measured (*n* in graph order: 14, 12, 13). Bars: mean values. Error bars: standard error of mean. ***p < 0.001, ****p < 0.0001 one-way ANOVA. **C** LIPE knockdown decreases monounsaturated FAs. FA profiles of cells treated per (**B**). Baseline abundance (Abd) indicated by green/gray bar, calculated on relative amount of each FA species in control cells. Red/blue heatmap is a representation of a given FA species. Saturated/unsaturated status indicated by white/black bars. **D** LIPE inhibition (LIPEi) decreases αS inclusion formation in a pre-treatment paradigm. M17D/αS-3K::YFP cells were incubated (16 h) with LIPE inhibitor 13g. αS expression induced and number of inclusions measured after 24 h of induction (*n* in graph order: 13, 14, 14, 13, 14). Bars: mean values. Error bars: standard error of mean. *p < 0.05, ****p < 0.0001 by one-way ANOVA. See Supplementary Fig. 2A for associated viability data. **E** LIPEi decreases αS inclusion formation in a pre-treatment paradigm. Microscopy images show decreased inclusions (green channel) upon treatment with 10 μM 13g. Images representative of ≥10 images in **D**. **F** LIPEi decreases αS inclusion formation in a treatment paradigm. αS induced (20 h), cells treated with LIPEi 13g (16 h). Bars: mean values. Error bars: standard error of mean. ***p < 0.001 by one-way ANOVA (*n* in graph order: 14, 14, 14). Supplementary Fig. 2B: associated viability data. **G** LIPEi reduces pSer129 αS. Cell lysates were immunoblotted to quantify pSer129 αS, total αS, GAPDH (*n* = 6). Bars: mean values. Error bars: standard deviation. **p < 0.005, ***p < 0.001 unpaired *t*-test. **H** LIPEi increases αS T:M ratio. M17D/αS-3K incubated (48 h) 20 μM 13g LIPE inhibitor subjected to 0.5 mM DSG crosslinking. Cell lysates immunoblotted to quantify αS14, αS60, and DJ-1 (crosslinking control). Control (DMSO) *n* = 12, LIPEi *n* = 8. Bars: mean values. Error bars: standard deviation. *p < 0.05 unpaired *t*-test. *non-specific band^112^. See Supplementary Fig. 2G. Statistics: GraphPad Prism 8.

We recently established that combined genetic knockdown of adipose triglyceride lipase and LIPE homologs rescued αS cytotoxicity in a yeast model^23^. That work also showed that elevated expression of wt or fPD E46K αS increased monounsaturated FA biosynthesis, predominantly 18:1n9 oleic acid (OA), and this escalated the formation of round, αS-positive (αS+), vesicle-rich cytoplasmic inclusions in neural cells^23^. In accord, SCD inhibition, which decreases monounsaturated FA levels, reduced the αS inclusion phenotype. This work principally addressed FAs generated through synthesis pathways. FAs can also be generated via degradation pathways (lipase activity) (Fig. 1A), and this could provide a distinct approach to treating PD and other synucleinopathies. To examine lipase activity as a potential PD therapeutic target, we first knocked down LIPE, the rate-limiting enzyme for neutral lipid degradation (Fig. 1A), in a cellular model of PD-like αS inclusion formation^42^. Amplifying the fPD E46K mutation (KTKEGV in repeat motif #4 becomes KTKKGV) by inserting analogous E→K mutations in the two adjacent KTKEGV motifs (E35K+E46K+E61K, called αS-3K) induces multiple, round αS+ cytoplasmic inclusions of clustered vesicles in neural cells^42,43^. These αS-3K inclusions have been shown to respond to known modifiers of wt αS neurotoxicity^23,33,44^. We treated M17D human neuroblastoma cells expressing αS-3K::YFP with two different shRNAs targeting LIPE. Knockdown decreased αS inclusion formation by at least 40% when compared to the control (Fig. 1B) without eliciting toxicity (Supplementary Fig. 1A, B). To assess whether decreasing LIPE induced FA alterations, shRNA-treated and control αS-3K::YFP cells were profiled for FAs by gas chromatography (GS) focused on phospholipid-incorporated FA. LIPE knockdown resulted in decreased levels of 18:1n9, 16:1n9, 16:0, and 18:1n7. The decrease in 18:1n9 was particularly noteworthy, given the high abundance of that FA (Fig. 1C and Supplementary Fig. 1C, D). From our previous work, we know that reducing monounsaturated FAs rescues several PD-relevant phenotypes^23^. Our new findings suggest that LIPE inhibition decreases the cellular pool of monounsaturated FAs and therefore could be a distinct therapeutic approach.

### Pharmacological inhibition of LIPE decreases αS-positive cytoplasmic inclusions

As a complementary approach, we assessed the role of the neutral lipid lipase LIPE in αS inclusion formation using pharmacological inhibitors. Neutral lipid lipase inhibitors (Table 1) were applied at multiple doses for 16 h before inducing αS expression in the αS-3K::YFP M17D cells. Inhibition of lipases with either of two broad-range inhibitors (Orlistat and CAY10499) strongly lowered αS+ cytoplasmic inclusions. Orlistat dose-dependently reduced inclusions while CAY10499 did not show a distinct dose dependency at the three doses tested (Supplementary Fig. 1E, F). These inhibitors were valuable for proof of principle but can regulate multiple lipases. Hence, we next tested LIPE-specific inhibitors. Pre-treatment with the LIPE inhibitor 13g^45^ dose-dependently prevented αS+ cytoplasmic inclusions (Fig. 1D, E) without eliciting cytotoxicity (Supplementary Fig. 2A). To evaluate 13g in a treatment mode, we induced αS expression for 24 h, then treated with this LIPE-specific inhibitor and assayed inclusions 16 h later. αS+ inclusion formation was significantly decreased by 10 μM treatment (Fig. 1F) without impacting cell viability (Supplementary Fig. 2B). LIPE inhibition by 13g resulted in reduction of similar species, e.g., 18:1n9, 18:1n7, 16:1n7, as seen by LIPE knockdown (Supplementary Fig. 1G). Similarly, pre-treatment (Supplementary Fig. 2C, D) or treatment (Supplementary Fig. 2E) with another LIPE-specific inhibitor, BAY^46^, dose dependently decreased αS inclusion formation, again without eliciting toxicity (Supplementary Fig. 2C). FA profiling via GS of the BAY-treated cells revealed a decrease in the same FAs identified for the genetic LIPE knockdown (above), with 18:1n9 being the most prominently decreased (Supplementary Fig. 2F). Based on these genetic and pharmacological analyses, LIPE inhibition can downregulate cytoplasmic inclusions in PD-relevant cellular models. We next sought to establish whether FA alteration by LIPE inhibition impacts phosphorylated αS, a biochemical hallmark of PD.

### Pharmacological inhibition of LIPE decreases phosphorylated αS and increases the native αS T:M ratio in αS-3K neural cells

In humans, increased αS phosphorylation, especially at Serine 129 (pSer129 αS), occurs in the LBs and neurites of LBD^47^ and fPD^48^ and is associated with greater neuropathological severity in idiopathic PD^49,50^. We asked whether LIPE inhibition changes the pSer129:total αS ratio. For initial screening, we used the M17D human neuroblastoma cells that constitutively express αS-3K. Treatment with the 13g LIPE-specific inhibitor significantly decreased the pSer129:total αS ratio (Fig. 1G).

αS conformation and assembly state influence cytotoxicity such that shifting the levels of physiological α-helical tetramers toward the more aggregation-prone monomers leads to αS cytoplasmic inclusions, hyperphosphorylation, and neurotoxicity^42,43,51,52^. Conversely, a reduction in αS+ inclusions in neural cells expressing E46K-derived mutations like 3K is often accompanied by increased native αS tetramers^23,33^. It is known that protein conformation can be impacted by transient interactions with FAs and lipids^53–55^. Therefore, we used the constitutively expressing αS-3K cells to investigate the impact of LIPE inhibition on αS assembly state. Specific inhibition of LIPE by 13g significantly increased the ratio of tetrameric to monomeric αS conformers (T:M) in the cytoplasm: the decrease in LIPE activity was associated with increased T:M ratios (60/14 kDa, 80/14 kDa, and 60 + 80/14 kDa), as quantified by intact-cell disuccinimidyl glutarate (DSG) crosslinking^52^ (Fig. 1H and Supplementary Fig. 2G). To determine the phospholipid-incorporated FA changes induced by the 13g LIPE inhibitor, αS-3K M17D cells treated with 13g were compared to untreated control cells by GS. The monounsaturated FAs 18:1n9, 18:1n7, and 16:1n9 were each significantly reduced upon 13g treatment (Supplementary Fig. 3A), in keeping with our findings for genetic LIPE knockdown and BAY inhibitor treatment.

### LIPE inhibition reduces monounsaturated FA and phosphorylated αS and increases αS T:M in αS E46K-expressing neurons

**Fig. 2:**
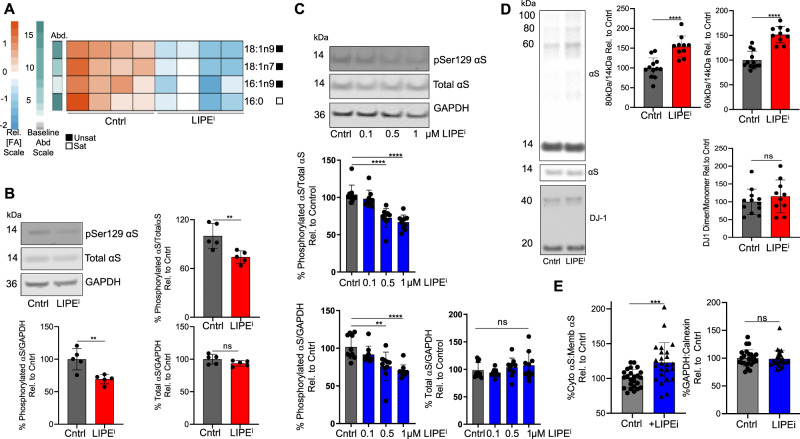
Inhibiting LIPE activity reduces pSer129 αs and increases native αs multimers. **A** LIPEi (13g) primarily decreases monounsaturated FAs in αS E46K-expressing cells. M17D/αS E46K (untagged αS E46K expressed constitutively) incubated with 10 μM 13g (20 h) (Supplementary Fig. 3C LDH data). Baseline abundance (Abd) indicated by green/gray bar, calculated on relative amount of each FA species in control cells. Red/blue heatmap is a representation of a given FA species. Saturated/unsaturated status indicated by white/black bars. **B** LIPEi reduces pSer129 αS in fPD αS E46K-expressing human neurons. αS E46K-expressing neurons were treated with 2 μM 13g. Cell lysates were immunoblotted to quantify pSer129 αS, total αS, and GAPDH (*n* = 5). Bars: mean values. Error bars: standard deviation. **p < 0.01 unpaired *t*-test. See also Supplementary Fig. 2H. **C** LIPEi dose-dependently reduces pSer129 αS in fPD αS E46K-expressing-induced neurons. αS E46K-expressing neurons treated with multiple doses of LIPEi BAY. Cell lysates were immunoblotted to quantify pSer129 αS, total αS, and GAPDH. (n in graph order: 10, 9, 10, 10). Bars: mean values. Error bars: standard deviation. **p < 0.01, ****p < 0.0001 by one-way ANOVA. **D** LIPEi increases αS T:M ratio in E46K-expressing neurons. Neurons expressing αS E46K were treated with 20 μM 13g (or DMSO control) subjected to 0.5 mM DSG crosslinking. Cell lysates were immunoblotted to quantify αS14, αS60, and DJ-1 (crosslinking control). Control (DMSO) *n* = 12, LIPEi *n* = 10. Bars: mean values. Error bars: standard deviation. ****p < 0.0001 unpaired *t*-test (GraphPad Prism 8). See also Supplementary Fig. 3F. **E** LIPEi (1 μM BAY) redistributes membrane αS to the cytosol in E46K neurons. *n* = 24. Sequential extraction performed per^77^. GAPDH (cytosol) and calnexin (membrane) used as extraction and normalizing controls. Bar charts quantify ratio differences of cytosolic:membrane αS and GAPDH:calnexin extraction controls between control and LIPEi-treated neurons. Bars: mean values. Error bars: standard deviation. ***p < 0.005 unpaired *t*-test. Statistics: GraphPad Prism 8.

LIPE inhibition significantly rescued PD-relevant phenotypes in the αS-3K model, an exacerbation of the E46K mutation. It was important to establish the LIPE inhibition-induced FA alterations in cells expressing the fPD-causing E46K clinical mutation. Constitutively expressing αS E46K M17D neural cells were treated with 13g LIPE inhibitor (10 μM) for 24 h, and phospholipid-incorporated FA were quantified relative to control cells by GS. The 13g-treated cells had reduced levels of 18:1n9, 16:1n9, 18:1n7, and 16:0 (Fig. 2A), recapitulating the earlier findings in the αS-3K M17D cell model (Fig. 1C and Supplementary Figs. 1D, 2F, and 3B). In the E46K αS M17D cells, LIPE inhibition by 13g treatment reduced pSer129 αS without changing total αS levels or eliciting toxicity (Supplementary Fig. 3C, D).

We next sought to replicate these findings in iPSC-derived human neurons transduced to express the fPD αS E46K mutation. The neurons were treated with 13g LIPE inhibitor (2 uM), causing a significant decrease in phosphorylated αS (whether normalized to total αS or to GAPDH) (Fig. 2B and Supplementary Fig. 2H). Next, we tested a structurally distinct LIPE-specific inhibitor, BAY, and found that it dose-dependently reduced phosphorylated αS in the E46K neurons (whether ratioed to total αS or GAPDH) without eliciting toxicity (Fig. 2C). Profiling of phospholipid membrane-integrated FA in BAY-treated vs. control cells showed decreases in multiple FAs, with the most prominent reductions in 18:1n9, 20:1n9, and 18:1n7 (Supplementary Fig. 3E). The fPD E46K mutation is known to decrease αS native tetramers and increase monomers^43^. 13g LIPE inhibition significantly restored the T:M αS ratio in the human neurons (Fig. 2D and Supplementary Fig. 3F). Increased tetrameric αS results in more αS localized in the cytosol^23,33^. Given that the additional positive charge in E46K αS increases αS:membrane interaction (i.e., lower αS cyto:αS memb)^18,56–61^ and that increased αS:membrane interaction is relevant to PD (cytosolic αS is less toxic than membrane αS)^56,62^, we assayed whether treatment of the E46K neurons with LIPEi would redistribute E46K αS from the membrane to the cytosol. Using in situ sequential extraction, LIPEi subtly but significantly increased cytosol:membrane αS ratio in E46K-expressing neurons (Fig. 2E).

### LIPE knockdown in a *Caenorhabditis elegans* model of αS rescues dopaminergic neuron loss

**Fig. 3:**
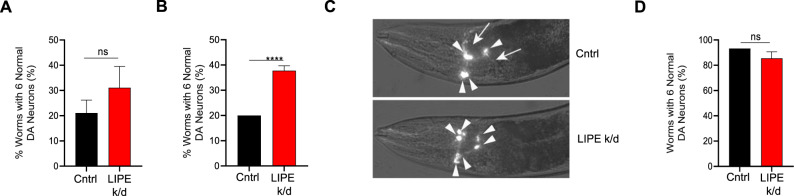
Reducing LIPE activity reduces dopaminergic neuron degeneration in an αS dopaminergic neuron degeneration *C. elegans* model. **A** *hosl-1* knockdown cell non-autonomously in *C. elegans* for one generation in dopaminergic neurons. Dopaminergic neurodegeneration in control EV RNAi and *hosl-1* RNAi in animals (P_*dat-1*_::αS + P_*dat-1*_::GFP animals) following one generation of RNAi exposure. Bars represent mean values of *N* = 3. Error bars indicate standard deviation. **B** *hosl-1* knockdown cell non-autonomously in *C. elegans* protects against αS-induced dopaminergic neurodegeneration. Dopaminergic neurodegeneration in control EV RNAi and *hosl-1* RNAi in animals (P_*dat-1*_::αS + P_*dat-1*_::GFP animals) following two generations of RNAi exposure. Data are reported as percentage of worms that display all six normal dopaminergic neurons; *n* = 30 adult worms for each of three independent experiments (total of 90 worms) for each condition. Bars represent mean values of *N* = 3. Error bars indicate standard deviation. ****p < 0.0001 by Student’s *t*-test (GraphPad Prism 8). **C** Representative images of dopaminergic neurons from *C. elegans* expressing P_*dat-1*_::αS + P_*dat-1*_::GFP. Intact dopaminergic neurons are indicated by arrowheads. Neurons showing signs of degeneration in the control (EV RNAi) are indicated by arrows while all six anterior dopaminergic neurons are protected following *hosl-1* RNAi. **D** *hosl-1* knockdown cell non-autonomously in *C. elegans* in GFP-only dopaminergic neurons. Dopaminergic neurodegeneration in control EV RNAi and *hosl-1* RNAi in animals (P_*dat-1*_::GFP animals) following two generations of RNAi exposure. Data are reported as percentage of worms that display all six normal dopaminergic neurons; *n* = 30 adult worms for each of three independent experiments (total of 90 worms) for each condition. Bars represent mean values of *N* = 3. Error bars indicate standard deviation (GraphPad Prism 8).

Given the consistent benefits of LIPE knockdown or inhibition in several cellular models described above, we assessed whether reducing LIPE could alter dopaminergic neurodegeneration in vivo. We had previously shown that decreasing OA synthesis by genetically reducing SCD in a wt αS-expressing *C. elegans* model decreased dopaminergic neurodegeneration^23,34^. In an analogous fashion, we knocked down *hosl-1*, the *C. elegans* homolog of LIPE, using RNAi in worms sensitive to RNAi in most tissues and expressing wt human αS under control of a dopamine transporter-specific promoter [P_*dat-1*_::αS + P_*dat-1*_::GFP]. Adult hermaphrodites were used to obtain a synchronized population of embryos. The F1 progeny were raised on bacteria expressing *hosl-1 KD* or empty vector (EV) RNAi and analyzed for dopaminergic neurodegeneration on day 8 post hatching. Worms exposed to *hosl-1* RNAi knockdown (LIPE k/d) showed less neurodegeneration than controls. While only 20% of control animals displayed a normal phenotype, 31% displayed normal neurons upon *hosl-1* knockdown (LIPE k/d); however, this was not statistically significant (Fig. 3A and Supplementary Fig. 3G). It is known that some targets are more resistant to RNA silencing compared to others and that two generations of RNAi may be needed to remove both cytoplasmic and nuclear RNAs. In this regard, exposing multiple generations of *C. elegans* to RNAi can effectively knockdown these targets^63^. Therefore, we exposed the worms for two generations to *hosl-1* RNAi (LIPE k/d) or EV treatment. Following this two-generational exposure paradigm, *hosl-1* RNAi worms displayed significantly less αS-induced dopaminergic neurodegeneration than controls (Fig. 3B,C and Supplementary Fig. 3H). Only 20% of control worms displayed a normal phenotype, while 38% of *hosl-1* RNAi (LIPE k/d) worms displayed a normal phenotype. As a control, we examined our GFP-only strain, which has no significant neurodegeneration. Following two generations of EV or *hosl-1* RNAi (LIPE k/d), there was no neurodegeneration in these animals, and importantly, hosl-1 knockdown (LIPE k/d) did not impact the percent of worms with 6 normal dopaminergic neurons (Fig. 3D and Supplementary Fig. 3I). As very little knockdown occurs in neurons using this method^64^, we can conclude that FA reduction via knockdown of the *C. elegans* LIPE homolog *hosl-1* represses αS-induced degeneration of dopaminergic neurons non-cell autonomously in vivo.

### PD patient-derived αS triplication neurons have abnormal pSer129 αS and UPR levels and decreased αS T:M phenotypes, all of which are reversed by LIPE inhibition

**Fig. 4:**
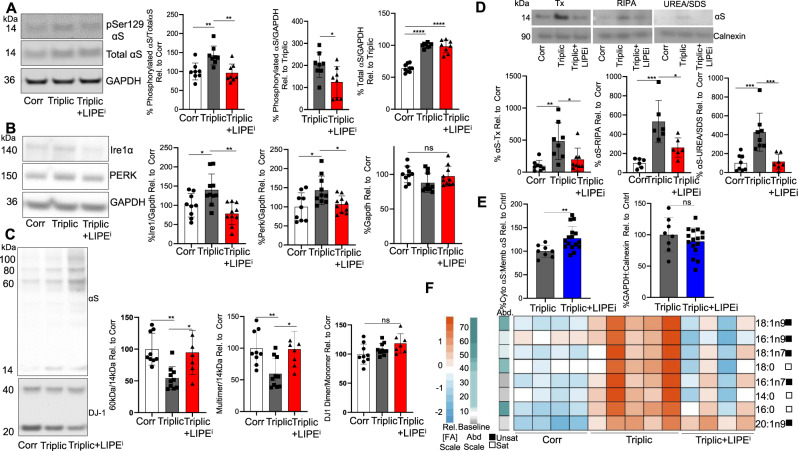
Inhibiting LIPE activity reduces pSer129, UPR defects and increases native αS T:M ratio in patient-derived iPSC αS triplication neurons relative to isogenic corrected neurons. **A** Patient-derived αS triplication neurons have increased pSer129 αS relative to isogenic corrected neurons. LIPEi restores pSer129 αS levels in patient-derived αS triplication neurons to that of isogenic control neurons. Cell lysates were immunoblotted to quantify pSer129 αS, total αS, and GAPDH. While pSer129 αS bands appear faint, results are reliably and reproducibly quantifiable by LI-COR imaging analysis. *n* = 8. *p < 0.05, **p < 0.01 one-way ANOVA. **B** LIPEi reduces UPR defects of patient-derived αS triplication neurons to that of isogenic control neurons. Cell lysates were blotted to quantify UPR master regulators Ire1α and PERK and GAPDH (*n* in graph order: 9, 10, 10). *p < 0.05, **p < 0.01 one-way ANOVA. **C** Patient-derived αS triplication neurons have decreased αS T:M ratio relative to isogenic corrected neurons. LIPEi increases αS T:M ratios of patient-derived αS triplication neurons to that of isogenic control neurons. Isogenic corrected and αS triplication with and without 10 μM 13g subjected to 0.5 mM DSG crosslinking. Cell lysates immunoblotted to quantify αS14, αS60, and DJ-1 (crosslinking control) (*n* in graph order: 9, 10, 7). Multimers/14 kDa:total 60 kDa + 80 kDa + 100 kDa/14 kDa. *p < 0.05, **p < 0.01 one-way ANOVA. **D** Patient αS triplication neurons have more insoluble αS than isogenic corrected neurons. This is reversed by LIPEi. Cell pellets of isogenic corrected neurons, patient-derived αS triplication neurons with and without LIPEi were sequentially extracted using triton-X (n:8), RIPA (n:6), and UREA + SDS (*n* in graph order: 8, 8, 7) buffers. *p < 0.05, **p < 0.01, ***p < 0.005 by one-way ANOVA (GraphPad Prism 8). **E** LIPEi (1 μM BAY) redistributes membrane αS to the cytosol in αS triplication neurons. Sequential extraction per^77^. GAPDH (cytosol) and calnexin (membrane): extraction and normalizing controls. αS triplication neurons (n:8), LIPEi-treated αS triplication neurons (n:16). **p < 0.01 unpaired *t*-test. **F** LIPEi (5 μM 13g) restores the FA profile of αS triplication neurons to that of isogenic corrected neurons. Baseline abundance (Abd) indicated by green/gray bar, calculated on relative amount of each FA species in control cells. Red/Blue heatmap is a representation of a given FA species. Saturated/unsaturated status indicated by white/black bars. Statistics: GraphPad Prism 8. All bars: mean values. All error bars: standard deviation.

E46K αS or wt αS overexpression models are premised on the mutated or excess αS interacting more or differently with vesicular phospholipid membranes. Importantly, we next analyzed iPSC-derived patient neurons reflecting the endogenous αS state instead of overexpression, in order to assess αS homeostasis in a cellular model of humans with PD. Triplication of the wt αS locus causes an aggressive, early-onset form of PD^3,65–68^. To test whether triplication of wt αS alters phosphorylated αS, we differentiated the patient-derived αS triplication neurons and their isogenically corrected control neurons to DIV 25 and assayed αS pSer129 levels as a function of total αS or GAPDH. The triplication neurons had significantly more phosphorylated αS relative to total αS or GAPDH than the isogenically corrected neurons (Fig. 4A). This is in keeping with a previous finding that αS triplication neurons have increased pSer129 relative to other wt lines^68^. We previously reported that SCD inhibition reduces phosphorylated αS in several wt αS overexpression cell models^23^. As proof of principle to establish the impact of SCD inhibition on endogenous αS, we asked whether SCD inhibition would alter phosphorylated αS in the patient-derived αS triplication neurons. Treatment with the SCD inhibitor 5b^35,69^ lowered the pSer129:total αS ratio in the αS triplication neurons back to that of the corrected neurons (Supplementary Fig. 4A), most prominently reducing 18:1n9 (Supplementary Fig. 4I). We then examined the impact of inhibiting LIPE (using 13g) on pSer129 αS levels and found that it reduced pSer129αS:total αS of the triplication neurons back to that of the corrected neurons (Fig. 4A). Similarly, the structurally distinct LIPE inhibitor BAY also reduced pSer129 αS:total αS of the triplication neurons toward that of the corrected neurons (Supplementary Fig. 4B).

Defects in vesicle trafficking pathways associated with the ER and ER stress have been associated with PD^70–72^. Specifically, patient iPSC-derived neurons with triplication of the αS locus have increased ER stress, signified by an increased UPR relative to isogenic corrected neurons^73^. Evidence of an increased UPR has also been described in postmortem PD brains relative to controls^73^. We sought to assess whether our lipid-related PD therapeutic approaches impact the UPR pathway in αS triplication patient neurons. As expected, the αS triplication patient-derived neurons had increased Ire1α and PERK (two UPR master regulators) relative to their isogenic control neurons (Fig. 4B). This pattern was reversed by SCD inhibitor treatment (compound 5b), providing proof of principle that an FA-related drug known to reverse PD-like phenotypes alters the UPR phenotype of patient-derived neurons (Supplementary Fig. 4C). Likewise, LIPE inhibitor treatment decreased Ire1α levels in the patient triplication lines to levels observed in the corrected lines. (Fig. 4B). We also assayed PERK levels and found that LIPE inhibition reduced the level of this UPR master regulator in the patient-derived αS triplication neurons to that of the control neurons (Fig. 4B).

Like αS missense mutations, triplication or duplication of the αS gene also causes fPD^3^, but whether this trait alters the T:M equilibrium has not previously been established. We assessed whether the excess of endogenous wt αS in a triplication family alters the T:M ratio in iPSC-derived neurons relative to an isogenic corrected line. Triplication of the αS locus in the patient-derived neurons resulted in a decrease in T:M conformer ratio (whether measured as 60/14 kDa or 60 + 80 + 100/14 kDa ratio) of the endogenous αS, whereas the endogenous DJ-1 dimer:monomer ratio measured simultaneously in the same cells was unchanged (Fig. 4C). Importantly, inhibition of LIPE using two independent inhibitors (13g; BAY) restored the T:M ratio to that of the corrected neurons, while the DJ-1 control was unaffected (Fig. 4C and Supplementary Fig. 4D, E).

Insoluble αS aggregates are a key neuropathological feature of PD. We observed significantly more membrane-associated as well as highly insoluble αS upon sequential extraction (Triton-X100, then RIPA, then UREA+SDS fractions)^74–76^ in αS triplication than corrected neurons (Fig. 4D). In general, the triplication neurons have approximately twice as much total cellular αS as their corrected line [e.g., Supplementary Fig. 6C (WB) and Supplementary Fig. 5C (immunocytochemistry [ICC])]. In contrast, the increases in the membrane-associated αS (Triton and RIPA fractions) and highly insoluble αS (UREA+SDS fraction) in the triplication neurons were approximately four times that of the corrected line (Fig. 4D). Importantly, treatment of the triplication neurons with LIPEi reduced the membrane-associated and insoluble αS to the control cell levels (Fig. 4D). Having thus established that the rise in insoluble αS in triplication neurons is reversed by LIPEi, we asked whether this was accompanied by an increase in cytosolic αS. Triplication of the αS locus does not alter the relative cytosol:membrane ratio of αS (Supplementary Fig. 6B). However, due to the triplication, there is more total αS and therefore more αS at membranes (Supplementary Fig. 5C). Increased αS:membrane interaction may be relevant to the PD process: cytosolic αS is less toxic than membrane αS^56,62^. To establish whether the LIPEi rescue of the abnormally elevated insoluble αS in triplication neurons (Fig. 4D) increases cytosolic αS, we used an orthogonal fractionation method: in situ sequential extraction^77^. LIPEi treatment of the triplication neurons shifted αS toward the cytosolic extract (Fig. 4E).

The dynamics of cytosol:membrane distribution of αS is impacted by membrane composition^23,33^, and LIPEi treatment might impact membrane composition and LDs. To examine whether αS localization changes in response to LIPEi treatment, we performed ICC for both αS and LDs on the PD triplication (Tripl) and isogenic control (Corr) human neurons treated with the LIPE inhibitor (Supplementary Figs. 5 and 6). As expected, there is a ~2-fold increase in the level of total αS immunoreactivity between Corr and Tripl as expected, and LIPEi does not significantly change this (Supplementary Fig. 5C). Importantly, an analysis of colocalization of LD (670 nm)/αS (570 nm) (Supplementary Fig. 5D) and of αS (570 nm)/LD (670 nm) (Supplementary Fig. 5E) using Mander’s Coefficient showed no significant re-localization of αS to LDs in response to LIPEi treatment.

### Triplication of the αS locus increases FAs, and LIPE inhibition alleviates this imbalance

In prior work, we observed that αS triplication upregulates the neutral lipid pathway components DG and TG, suggesting an increase in FA levels^23^. We also noted that excess wt αS results in increased monounsaturated FAs in several cell models overexpressing exogenous αS, including human iPSC-derived cortical neurons and primary rat cortical neurons^23^. To establish FA profiles in the patient-derived αS triplication neurons, we assayed the neurons at DAY25 post differentiation and focused specifically on phospholipid-incorporated FAs. As expected based on our previous findings of DG and TG increases, we observed an overall increase in FAs in the αS triplication neurons relative to the corrected line. The most prominent significantly increased FAs included monounsaturated FA: 18:1n9, 16:1n9, and 18:1n7 (Fig. 4F). These three monounsaturated FAs were reduced when the αS triplication neurons were treated with the LIPE inhibitors 13g (Fig. 4F) or BAY (Supplementary Fig. 4F). Neither treatment elicited toxicity (Supplementary Fig. 4G, H). The increased incorporation of unsaturated FA into membrane phospholipids is biologically important, as these increase membrane fluidity, potentially altering αS interactions with curved vesicle membranes.

### 16:1- and 18:1-containing phospholipid classes are reduced by LIPEi

**Fig. 5:**
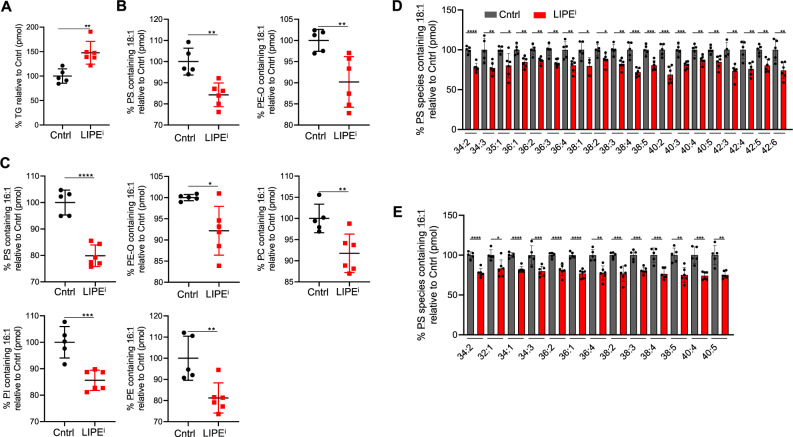
LIPEi (13g) reduces FA species 18:1 and 16:1 in phospholipid classes PS, PI, PE, PE-O. **A** TG are increased in patient-derived αS triplication neurons upon LIPEi. Total triglycerides (TG) (pmol) were measured by mass spectrometry lipid profiling in patient-derived αS triplication neurons untreated (Cntrl) or treated with 5 μM 13g. Middle line: mean values (*n* in graph order: 5, 6). **p < 0.01 unpaired *t*-test. **B** PS and PE-O classes containing 18:1 species are decreased upon LIPEi. PS and PE-O classes containing 18:1 FA species analyzed by mass spectrometry lipid profiling in patient-derived αS triplication neurons untreated (Cntrl) and with 5 μM 13g. Data for species containing 18:1 FA analyzed here. Middle line: mean values. Error bars: standard deviation (*n* in graph order: 5, 6). **p < 0.01 unpaired *t*-test. **C** PS, PE-O, PE, PC, PI classes containing 16:1 species are decreased upon LIPEi. Lipid classes containing 16:1 FA species analyzed by mass spectrometry lipid profiling in patient-derived αS triplication neurons untreated (Cntrl) and with 5 μM 13g. Data for species containing 16:1 FA analyzed here. Middle line: mean values. Error bars: standard deviation (*n* in graph order: 5, 6). **p < 0.01 unpaired *t*-test. **D** PS containing 18:1 species are reduced in patient-derived αS triplication neurons upon LIPEi treatment. PS (pmol) was measured by mass spectrometry lipid profiling in patient-derived αS triplication neurons untreated (Cntrl) and treated with 5 μM 13g. Data for species containing 18:1 FA were analyzed and reported here. Y-axis is % PS species relative to the control. PS species are listed on the *x*-axis. Bars: mean values. Error bars: standard deviation (*n* untreated samples = 5, except *n* = 4 for untreated 42:3 and *n* = 6 for all treated samples except *n* = 3 for treated 38:1). *p < 0.05, **p < 0.01, ***p < 0.001, ****p < 0.0001 unpaired *t*-test. **E** PS containing 16:1 species are reduced in patient-derived αS triplication neurons upon LIPEi treatment. PS (pmol) measured by mass spectrometry lipid profiling in patient-derived αS triplication neurons untreated (Cntrl) and treated with 5 μM 13g. Data for species containing 16:1 FA analyzed here. Bars: mean values. Error bars: standard deviation (*n* untreated samples = 5, *n* treated samples = 6). *p < 0.05, **p < 0.01, ***p < 0.001, ****p < 0.0001 unpaired *t*-test. Statistics: GraphPad Prism 8.

LIPE inhibition alters FA incorporation into phospholipids, mostly but not exclusively decreasing unsaturated FA incorporation, and this is associated with the rescue of PD phenotypes in patient-derived αS triplication neurons (above). To identify the phospholipids in which these FA alterations occur, we performed unbiased lipid profiling. We first assessed whether LIPE inhibition impacted total TG levels. As expected, treatment of the patient-derived αS triplication neurons with the LIPE inhibitors 13g or BAY increased total TG (Fig. 5A and Supplementary Fig. 7A), reducing the free FA pool available for incorporation into phospholipids. The LIPEi-treated cells with increased TG were significantly enriched for membrane-incorporated C18:1 (13g and BAY), and C16:1 (BAY) (Supplementary Fig. 8A, B). Both LIPE inhibitors also increased membrane-incorporated C16:0 in TG (Supplementary Fig. 8C, D). For phospholipid class analysis, we focused on lipid species containing the FAs 18:1 and 16:1, given their significant abundance in phospholipid membranes and reduction by LIPE inhibition. LIPEi (13g) decreased 18:1 FA species in phosphotidylserine (PS) and the plasmalogen phosphotidylethanolamine (PE-O) classes (Fig. 5B), while no differences were observed in phosphotidylglycerol (PG), phosphotidylcholine (PC), phosphatidic acid (PA) and phosphotidylethanolamine (PE) (Supplementary Fig. 7B). Similarly, the LIPE inhibitor BAY decreased 18:1 species in PS and PE-O and additionally in the PE and PI classes (Supplementary Fig. 7C). Analysis of phospholipid classes incorporating 16:1 species when treated with the 13g or BAY LIPE inhibitors showed a reduction in 16:1 species in PS, PE-O, PC, PI, and PE classes (Fig. 5C and Supplementary Fig. S7D). Interestingly, subspecies analysis of the PS species containing 18:1 or 16:1 that were reduced in the patient-derived neurons by 13g treatment showed similarities, e.g., PS 36:1, PS, 36:2, etc. (Fig. 5D, E). A number of these species have been shown to be increased in the cerebral cortex of PD patients^78^ (see Discussion). Importantly, our data suggest LIPE inhibition acts without altering total cellular lipid but by preserving FAs in TG form (increasing TG) and decreasing their incorporation into phospholipids.

### Targeting FA levels via synthesis and degradation pathways is additive, reducing αS inclusions and phosphorylation through cumulative decreases in 18:1n9 and 16:1n9

**Fig. 6:**
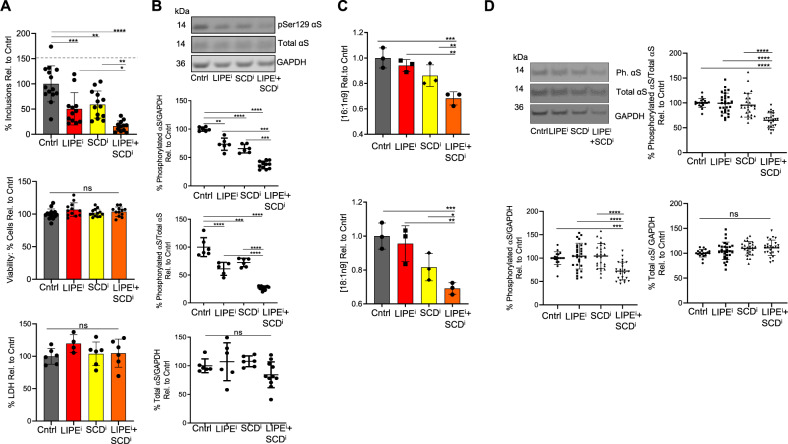
Co-regulating SCD and LIPE (synthesis and degradation) is additive in reducing αS inclusion formation and pSer129 αS through additive decreases in 18:1n9 and 16:1n9. **A** LIPEi and SCDi treatments are additive in reducing αS inclusion formation. M17D/αS-3K::YFP cells incubated (16 h) with 13g (20 uM) and SCD inhibitor 5b (0.05 uM). αS expression induced and number of inclusions measured after 24 h of induction (*n* in graph order: 14, 12, 13, 12). Bars: represent mean values. Error bars: standard error of mean. *p < 0.05, **p < 0.001, ***p < 0.001, ****p < 0.0001 one-way ANOVA. **B** LIPEi and SCDi treatments are additive in reducing pSer129 αS. Individually, LIPE inhibition (20 μM BAY) and SCD inhibition (20 μM HY19672) decrease the ratio of pSer129 αS:total αS in αS-3K cells, and the combination of inhibiting both targets is additive. Cell lysates were immunoblotted to quantify pSer129 αS, total αS, and GAPDH (*n* in graph order: 6, 5, 6, 12). Middle line: mean values. Error bars: standard deviation. **p < 0.01, ***p < 0.001, ****p < 0.0001 one-way ANOVA. **C** 18:1n9 and 16:1n9 are significantly reduced in the combined SCD and LIPE inhibition treatments relative to the control and relative to each treatment individually. M17D/αS-3K were treated with DMSO, HY19672 (SCDi) at 10 μM, BAY (LIPEi) at 10 μM or combined 10 µM HY19672 and 10 μM, BAY. Samples were harvested for FA analysis and analyzed by gas chromatography. Statistical analysis per Materials and methods section (*n* = 3 for all). Bars: mean values. Error bars: standard deviation. The two assayed FAs (18:1n9 and 16:1n9) with statistically significant differences in abundance between control, each individual treatment alone, and combined treatment are reported. **D** LIPEi and SCDi treatments are additive in reducing pSer129 αS in patient-derived αS triplication neurons. Individually, LIPE inhibition (2.5 μM BAY) and SCD inhibition (0.2 μM 5b) do not alter the ratio of pSer129 αS:total αS or pSer129 αS:GAPDH at suboptimal inhibitor doses used (note: these individual drug concentrations were chosen to facilitate the observation of additive effect). The combination of inhibiting both targets is additive. Cell lysates were immunoblotted to quantify pSer129 αS, total αS, and GAPDH (*n* in graph order: 19, 29, 26, 25). Middle line: mean values. Error bars: standard deviation. ***p < 0.001, ****p < 0.0001 one-way ANOVA. Statistics: GraphPad Prism 8.

We previously reported that reducing monounsaturated FA synthesis by inhibiting SCD reverses PD-relevant phenotypes in vitro and in vivo^23,35^. Our findings above now show that lowering cytoplasmic FA levels by inhibiting lipid degradation by LIPE can similarly reduce PD-relevant phenotypes caused by fPD E46K αS or excess wt endogenous αS (triplication). Both SCD and LIPE contribute to LD composition and influence protein interactions with phospholipid membranes. Hence, it is important to learn whether targeting both FA synthesis and degradation pathways could be additive in maintaining cellular FA equilibrium and thereby lessening PD-relevant outcomes. To establish whether combining SCD and LIPE inhibitors could be therapeutically useful, we assessed such inhibitors together for their impact on αS inclusion formation and phosphorylated αS. In accord with prior work^23^ and data presented above, SCD and LIPE inhibitors each independently reduced inclusion formation without cytotoxicity (Fig. 6A). When combined, inhibition of both SCD and LIPE was additive: this markedly and significantly reduced inclusions relative to controls and each treatment alone (Fig. 6A). To establish further relevance to PD, we assayed the impact of the combined approach on phosphorylated αS. Consistent with the inclusion data, simultaneous SCD and LIPE inhibition significantly reduced pSer129 αS relative to untreated and to each treatment alone (Fig. 6B). To gain deeper biochemical insight, we assayed the FA landscape of SCD and LIPE inhibitor treatments individually and together. Only two of the FAs we assayed were significantly decreased in the combination approach relative to control and each treatment alone: 18:1n9 and 16:1n9 (Fig. 6C). This result is again in keeping with our central hypothesis that decreasing monounsaturated FA is particularly beneficial. To establish whether SCD and LIPE inhibition would have an additive effect in PD patient neurons, we assayed pSer129 αS in the αS triplication neurons. Suboptimal concentrations of the LIPE and SCD inhibitors individually were used to enable observation of any additive effect of the combined inhibitor treatment on pSer129 αS. Importantly, combined treatment with LIPE and SCD inhibitors reduced pSer129 αS relative to total αS or to GAPDH as compared to the untreated patient-derived triplication neurons or neurons treated with each individual inhibitor (Fig. 6D). This finding in αS patient triplication neurons suggests that combination treatment that lowers monounsaturated FAs is a potential therapeutic strategy.

## Discussion

Growing evidence suggests PD and LBD are lipidopathies as well as proteinopathies (see Introduction). Excess or mutant αS expression results in altered FA metabolism, substantiating therapeutic targeting to re-establish FA metabolic homeostasis. Targeting FA synthesis via SCD inhibition has already proven successful in reversing or preventing PD-relevant phenotypes in multiple diverse models^23,32,35^ and has entered human trials, but this approach does not address the role of FAs generated via lipid degradation. In a rough analogy to AD, where a misfolded protein may accumulate from increased production or decreased clearance/degradation, here we investigated the lipid degradative process as a potential candidate for modifying synucleinopathy phenotypes.

A major target of lipid degradation is LDs (FA stores/reservoirs) generated via the neutral lipid pathway that functions in storing excess FAs as DG and TG to avoid cellular toxicity. Some patient brains have significant LD accumulation in postmortem studies^36,37^. This observation is in keeping with our identification of multiple components of the neutral lipids pathway (FA, DG, TG, and storage as LDs) as being elevated in response to excess or mutant αS^23^.

Here, we performed a targeted analysis of the neutral lipid degradation lipase pathway through a series of genetic and biochemical experiments (including two distinct LIPE-specific inhibitors) that provide new insights into the importance of FA homeostasis for PD-type synucleinopathy. We focused on phospholipid-incorporated FAs as regulating αS:membrane interactions believed to be important in the disease process. Our several models, including the fPD αS E46K mutant and patient-derived αS triplication neurons, converge on the effects of inhibiting LIPE, the rate-limiting neutral lipid lipase, as leading to a highly significant reversal of certain disease-relevant cellular phenotypes. The function of LIPE as a critical feature of FA mobilization and the catalyst of the first and rate-limiting step in TG hydrolysis (Fig. 1A) provides a potentially advantageous drug target. Genetic and pharmacological reductions of LIPE both rescued several PD-relevant phenotypes, including decreasing formation of round αS- and vesicle-rich cytoplasmic inclusions. Reducing LIPE enzymatic activity reversed PD-relevant phenotypes related to the E46K αS fPD mutation, including decreasing pSer129 αS, increasing the physiologic αS T:M ratio, and redistributing the abnormal membrane-bound αS associated with the E46K mutation to the cytosol. FA profiling confirmed that LIPE inhibition decreased unsaturated FAs that are incorporated into membrane phospholipids. This effect would decrease the fluidity of membranes with which αS interacts. Lipid profiling of patient-derived αS triplication neurons treated with LIPE inhibitors was analyzed, focusing on phospholipids containing 18:1 and 16:1 FAs. These species were reduced by two different LIPE inhibitors in almost all of the major phospholipid classes. This is somewhat expected, given the interconnected nature of phospholipid pathways and LIPE activity. A decrease was observed across phospholipid classes containing 16:1 and 18:1 FA species. Decreases in 18:1 species were observed in PS, PE-O, PI, and PE. Reduction in 16:1 species was observed in PS, PI, PE, PE-O, and PC, while significant changes were not discerned for PG or PA species. Changes in PC are noteworthy as it is the most abundant cellular membrane phospholipid^79^. In addition, PE comprises ~25% of mammalian phospholipids and ~45% of brain phospholipids, so alterations in this class are also important^80,81^. Furthermore, reduction in the plasmalogen class PE-O is notable given the role of plasmalogens in membrane structure and integrity, helping to determine protein:lipid interactions. The reduction of 18:1- and 16:1-containing PS species (36:1, 36:2, 38:5, 38:4, 38:3, 40:4) by LIPE inhibition is interesting given that these species have been identified as increased in the cerebral cortex of PD patients^78^. Both LIPE inhibitors resulted in very similar changes in phospholipids containing 18:1 and 16:1 in patient-derived αS triplication neurons, with BAY having the wider impact across lipid classes.

fPD E46K αS is more positively charged than wt αS and therefore interacts more with the relatively negatively charged phospholipid head groups in membranes. Increased αS binding to membranes is associated with disease-relevant phenotypes, especially at membranes with greater degrees of unsaturation (more fluid). Similarly, increased absolute amounts of αS at membranes result from the duplication or triplication of the αS locus in those families. Our findings demonstrate that altering phospholipid membrane composition using LIPEi can redistribute to a more cytosolic state the abnormal membrane-localized αS associated with the E46K and triplication mutations. Decreasing LIPE activity in such patient neurons relieved pSer129 αS accumulation, abnormal UPR, abnormal membrane-associated and highly insoluble αS (a PD-mimicking phenotype), and low T:M ratios, all features that have been observed in cellular and rodent models of PD.

The current work provides the first identification of certain PD-type biochemical changes in neurons of patients carrying a triplication of the αS gene. Specifically, we show that pSer129 αS, a marker of both familial and “sporadic” PD, accumulates in the αS triplication neurons. In addition, we answer a question posed after our discovery that all fPD-causing missense mutations in αS shift the T:M equilibrium toward excess monomers^43^: does this also occur in familial cases with excess αS gene dosage? The answer is yes: we show here that endogenous wild-type αS in human neurons from a triplication family have relatively less tetramers and more monomers. Importantly, this can be corrected by LIPE inhibition (Fig. 4C, D).

LIPE inhibition is a blunter approach to decreasing unsaturated FAs than is SCD inhibition, as LIPE cleavage generates both unsaturated and saturated FAs. Importantly, however, LIPE inhibition converted the FA profile of PD αS triplication neurons to that of corrected (wt) neurons (Fig. 4F), suggesting that this approach can help reverse the altered FA equilibrium that is associated with abnormal αS accumulation in neurons^23,35^. We note that: (i) LIPE has the greatest activity against the unsaturated FA 18:1n9^39,40^; (ii) LIPE inhibition is the most direct way of lowering FA pools generated through phospholipid degradation; (iii) our data indicate that LIPE reduction decreases more unsaturated than saturated FA; (iv) those saturated FAs that are decreased (e.g., stearic and palmitic) have not been associated with PD-relevant neuronal phenotypes on their own^23,33^; and (v) the greatest FA alterations we saw upon LIPE inhibition (e.g., 18:1n9) were those most abundant in phospholipid membranes, thus having potentially the most impact on membrane dynamics.

Importantly, SCD inhibition could prevent FA cytotoxicity and additional accumulation of LDs, but the decrease in overall brain FAs would be expected to generate compensatory FA release through LD degradation. Hence, co-regulating FA metabolism with SCD and LIPE inhibitors could be important for maintaining FA homeostasis in PD patients. We performed multiple assays in several cellular models using different combinations of both inhibitors to show that the simultaneous downregulation of FA synthesis and degradation can be additive. Roughly akin to increased generation and decreased clearance of amyloid β-protein each contributing to the pathogenesis of AD^82^, both FA production and FA degradation could be impaired in synucleinopathies and contribute to pathogenesis.

Why do monounsaturated FAs appear to be problematic in synucleinopathies? Higher cellular 18:1n9 levels mediate αS toxicity in part by increasing pSer129^33^ and elevating αS membrane binding^23^. The latter effect is in keeping with the accumulation of vesicle-rich inclusions that may result from excess fPD-mutant αS monomers at vesicle membranes^83,84^. Increased αS localization at membranes has been reported to confer neurotoxicity in numerous studies^56,85–89^. The decrease in endogenous αS tetramers that we document, including for the first time in αS triplication neurons, leads to more aggregation-prone monomers, associated with αS inclusions and neurotoxicity^23,83,90^. In a working hypothesis in keeping with our previous SCD inhibitor findings, we suggest two mechanisms for increased αS membrane binding in PD-mutant neurons: (1) enhanced membrane fluidity due to the increased incorporation of unsaturated fatty acyl side chains; and (2) possible binding of αS directly to monounsaturated FAs incorporated as fatty acyl side chains into a phospholipid membrane^19,20^. Our hypothesis proposes that high levels of monounsaturated FAs elevate αS membrane binding, thereby augmenting membrane-associated toxicity and ultimately resulting in excess monomeric αS polymerizing into higher-order aggregates (including fibrils)^86,88,91,92^. Our published data in^23^ and in more detail in^33^ show that unsaturated FAs increase αS association with membranes while saturated FAs do not (e.g., C18:1 does but C18:0 does not). Excess unsaturated FAs are known to increase membrane lipid packing defects. αS is known from many in vitro studies to preferentially bind to small, highly curved membranes that induce α-helical structure^93^. Our hypothesis is that natively unfolded monomers in the cytosol normally bind transiently to such curved membranes and become α-helical, and four helical monomers assemble into an energetically favored α-helical tetramer^51^, perhaps in the process of acquiring a limiting factor such as a small lipid. The tetramers come off the membrane and are localized almost entirely in the cytosol at a steady state^52^. A tetramer at some point loses its limiting factor and disassembles into unfolded monomers, and the cycle starts over. Excess monounsaturated FAs in membranes seem to interfere with the physiological tetramer:monomer recycling process and are associated with a decreased T:M ratio and excess free monomers that may ultimately assemble into abnormal insoluble aggregates; indeed, this may be reflected in the striking increase in the highly insoluble (Urea-SDS extractable) αS we observed in the triplication neurons, clearly in excess of the total αS increase conferred by the gene dosage. Excess unsaturated FAs are known to increase membrane fluidity, altering the sensitive and dynamic process of αS interaction with membranes and the proper formation of transient tetramers. In summary, both excess αS on membranes and an abnormal T:M equilibrium are related phenomena that represent a move away from the physiological state.

**Fig. 7:**
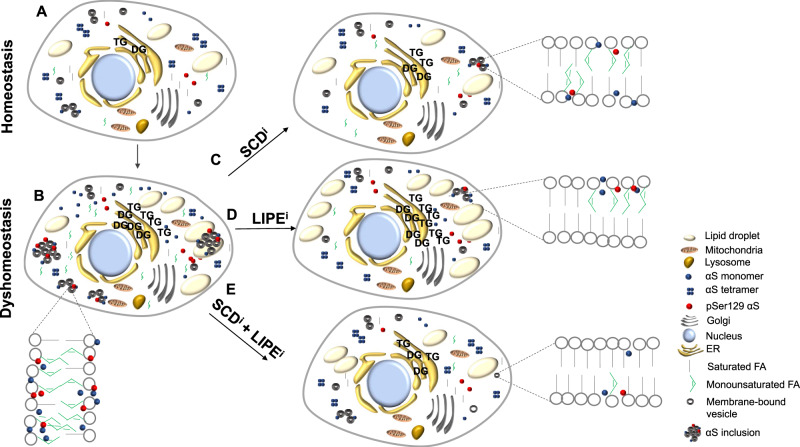
Summary model of FA therapeutic interventions and cellular αS biology primarily based on findings in this study and ref. ^23^. **A** Intact αS and FA homeostasis. An equilibrium of unfolded αS monomers (at membranes and in the cytosol) and physiological (cytosolic) α-helical tetramers exists. FA and lipids are in equilibrium. **B** αS dyshomeostasis. E46K αS or excess wt αS expression results in increased monounsaturated FA in phospholipid membranes and increased DGs, TGs, and LDs as the cell responds to evade cytotoxicity. The αS T:M ratio is reduced and levels of αS inclusions, pSer129 αS, and UPR are increased. **C** SCD inhibition. Inhibiting SCD reduces monounsaturated FAs incorporated into phospholipid membranes, decreasing DGs, TGs, and LDs. pSer129 αS levels, αS inclusions, UPR, and T:M αS are restored to homeostatic levels. **D** LIPE inhibition. Inhibiting LIPE reduces multiple FAs (primarily the most abundant monounsaturated FAs) and LDs accumulate^94^. pSer129 αS, αS inclusions, UPR, and T:M are restored to equilibrium. **E** Inhibition of both SCD and LIPE. Combined inhibition of SCD and LIPE reduces monounsaturated FA in phospholipid membranes further than that of either treatment individually. This reduction is accompanied by decreased αS inclusion formation and decreased pSer129.

Based on the data herein, we propose the following summary model to explain our findings (Fig. 7). With normal αS (non-mutant and non-excess) and normal lipid homeostasis, pSer129 αS and αS inclusions levels are low, and an equilibrium exists between unfolded monomers and α-helically folded physiological tetramers that are principally cytosolic, with some monomeric αS at membranes and in cytosolic pools^42,43,53,83,88^ (Fig. 7A). In contrast, excess wt αS (duplication/triplication) or mutant αS (e.g., E46K) can increase monounsaturated FAs in phospholipid membranes, as well as in DG, TG, and LDs to initially evade cytotoxicity^23^. The outcomes include increased αS monomers at membranes, clustering of vesicles bound with excess monomers^83,84^, increased pSer129 αS (Fig. 4A and Supplementary Fig. 4A), decreased αS T:M equilibrium (Fig. 4C), and enhancement of the UPR (Fig. 4B and Supplementary Fig. 4B, C)^42,43,73,83^ (Fig. 7B). Inhibiting SCD (Fig. 7C) decreases levels of membrane-incorporated unsaturated FAs, DG, TG and LD, pSer129 αS, αS inclusions, and αS at membranes, and it restores both T:M and UPR (Supplementary Fig. 4A, C and refs. ^23,33,35,90^). LIPE inhibition (Fig. 7D) maintains LD integrity^94^ and decreases phospholipid-incorporated unsaturated FAs (Figs. 1C, 2A, and 4F and Supplementary Figs. 1D, 2F, 3A, B, E, and 4F). Decreasing LIPE activity reduces αS inclusions (Figs. 1B, D–F and Supplementary Fig. 1A–C, E, F) and decreases pSer129 αS (Figs. 1G, 2B, C, and 4A), the αS T:M ratio (Figs. 1H, 2D, and 4C) and UPR to homeostatic levels (Fig. 4B). The additional benefit of LIPE reduction is the restoration of overall FA homeostasis in αS triplication neurons to that of the isogenic corrected neurons (Fig. 4F). Co-inhibiting SCD and LIPE (Fig. 7E) reduces monounsaturated FAs in phospholipid membranes (specifically 18:1n9 and 16:1n9) further than that of either treatment individually (Fig. 6C), balancing both synthesis and degradation processes. This balance results in additive decreases in αS inclusion formation and pSer129 αS (Fig. 6A–D).

We have demonstrated the benefit of LIPE activity reduction in αS gene dosage and missense mutations. These early-onset genetic forms of PD each accelerate disease, suggesting how wt αS as the key PD-related protein might behave in “sporadic” disease over longer time periods of aging. Hence, we believe targeting LIPE as a therapeutic point of intervention could be important not only for patients with fPD mutations but also in idiopathic PD. As SCD inhibitors are now being assessed in the clinic, it may be appropriate to consider LIPE inhibitors as functionally distinct but mechanistically related alternatives or complements for PD and LBD patients. It will be important to examine a broad range of PD-linked gene mutations and polymorphisms for which these candidate therapeutics could be applicable, including those “indirectly” impacting FA homeostasis, such as mutations in αS, as well as those PD genetic risk factors “directly” impacting FA equilibrium in their role in lipid metabolism, e.g., DGKQ and ELOVL7.

## Methods

### αS-3K and αS E46K-expressing neuroblastoma cells

#### Maintenance—neuroblastoma cells

Cells were grown in DMEM 1x (Dulbecco’s Modification of Eagle’s Medium) with 4.5 g/L glucose, L-glutamine, and sodium pyruvate (Corning 10-013-CV) containing 10% fetal bovine serum (Sigma F0926).

#### Inclusions assays—neuroblastoma cells

Cells were plated at a density of 10,000 cells per well in a 384-well plate. shRNA viruses (listed below) or inhibitors (Table 1) were added to wells for 24 h. Cells were induced using doxycycline for 24 h. Inclusions and mCherry signals were monitored for 24 h in the IncuCyte Zoom 2000 platform (Essen Biosciences). The impact of knockdown or pharmacological treatment was generally assayed by FA profiling instead of western blot or transcriptional readouts as more accurate monitoring of enzyme activity modification. See “FA sample preparation, profiling, and analysis (OmegaQuant)” for more detail. Abbrevations are listed in Table 2. Antibody and virus knockdown informationis outlined in Tables 3 and 4.

**Table 1:** Inhibitors.

Name	Target	Company	Ref code/Reference
BAY	LIPE	–	^46^
13g	LIPE	–	^45^
Orlistat	Multiple TG lipases	Cayman	10005426
CAY10499	Non-selective lipase inhibitor	Cayman	10007875

**Table 2:** Abbreviations.

αS	α-synuclein
PD	Parkinson’s disease
DSG	Disuccinimidyl glutarate
DG	Diglyceride
FA	Fatty acid
fPD	Familial Parkinson’s disease
GWAS	Genome-wide association studies
iPSC	Induced pluripotent stem cells
LBD	Lewy body dementia
LD	Lipid droplet
LIPE	Lipase E, hormone-sensitive type
PA	Phosphatidic acid
PC	Phosphotidylcholine
PE	Phosphotidylethanolamine
PE-O	Phosphotidylethanolamine-O (plasmalogen)
PG	Phosphotidylglycerol
PI	Phosphotidylinositol
PS	Phosphotidylserine
SCD	Stearoyl-coA desaturase
TG	Triglyceride

**Table 3:** Antibodies.

Protein	Secondary	Company	Ref code
αSynuclein (Syn1)	Mouse	BD	BDB610786
αSynuclein (4B12)	Mouse	ThermoFisher	MA1-90346
DJ-1	Rabbit	–	^111^
Phosphorylated αS (pSer129)	Rabbit	Abcam	ab168381
GAPDH	Rabbit	Abcam	ab9485
Ire1α	Rabbit	Novus Biologicals	NB100-2323
Perk	Rabbit	Abcam	ab229912

**Table 4:** Knockdown virus.

Gene/protein	Virus info from Sigma
*Control*	SHC016V
*LIPE*	TRCN0000427424 (NM_005357.2-1656s21c1) TRCN0000440990 (NM_005357.2-2129s21c1)

#### Phosphorylation assays—neuroblastoma cells

Cells were plated at a density of 100,000 cells per well in a 24-well plate. Cells were treated with drug for 48 h before harvesting. Briefly, cells were resuspended in TBS, transferred to Eppendorf tubes, and spun for 5 min at RT at 500 × g. Pellets were resuspended in 40 µL lysis buffer (TBS containing 0.7% triton-X, 0.1% tween-20, and 1× Halt Protease and Phosphatase Inhibitor (PPI) Cocktail (ThermoScientific 78440)) and lysed on ice for 20 min. Samples were spun at approximately 20,000 × g for 30 min at 4 °C.

Lysates were boiled at 95–100 °C with 1× NuPAGE LDS Sample Buffer (Invitrogen NP0007) for approximately 5 min and were then loaded into NuPAGE 4–12% Bis-Tris Midi 20-well gels (Invitrogen WG1402BX10). Gels were run initially at 60 V using 1× NuPAGE MES SDS Running Buffer (Novex NP0002) for approximately 30–45 min and then were run at 90 V until the blue dye ran off the gel. Gels were transferred to iBlot 2 NC Regular Stacks (Invitrogen IB2300) using the iBlot P0 protocol. Blots were fixed in 4% paraformaldehyde in TBS, blocked in Odyssey TBS Blocking Buffer (LI-COR 927-50000) for a minimum of 30 min, and left in primary antibody overnight at 4 °C on a shaker. Primary antibody (GAPDH: AB9485 [1:5000]; 4B12: MA1-90346 [1:1000]; MJF-R13: AB168381 [1:1000]) was diluted in Odyssey TBS Blocking Buffer with 0.2% Tween-20. The following day, blots were washed three times in TBS-T (TBS containing 0.1% Tween-20) for approximately 10 min each wash. Blots were put in the appropriate LI-COR secondary antibody diluted 1:5000 in Odyssey TBS Blocking Buffer with 0.2% Tween-20 and 0.01% SDS for 45 min at RT on a shaker. Blots were washed again in TBS-T for 10 min for a total of three washes. Blots were developed on “medium” using the LI-COR Odyssey CLx instrument. Bands were quantified using LI-COR Image Studio Lite software. The background for this quantification was set to “median” with a border width of three along all the borders.

#### Crosslinking—neuroblastoma cells

Cells were plated at a density of 100,000 cells per well. Cells were treated with drug for 48 h before harvesting. Briefly, cells were resuspending in PBS, transferred to Eppendorf tubes, and pelleted for 5 min at RT at 500 × g. Pellets were crosslinked in 0.5 mM DSG (ThermoScientific 20593) in PBS containing PPI for 30 min at 37 °C with gentle agitation. After that time, excess DSG was neutralized with 1 M Tris Hydrochloride (Fisher Scientific BP1757-100). Cells were lysed in a final concentration of 0.7% triton-X in TBS-T containing PPI on ice for 20 min. Samples were then spun at approximately 20,000 × g for 30 min at 4 °C.

Lysates were boiled at 95–100 °C with 1× NuPAGE LDS Sample Buffer (Invitrogen NP0007) for approximately 5 min and were then loaded into NuPAGE 4–12% Bis-Tris Midi 20-well gels (Invitrogen WG1402BX10). Gels were run initially at 60 V using 1× NuPAGE MES SDS Running Buffer (Novex NP0002) for approximately 30–45 min and then were run at 90 V until the blue dye ran off the gel. Gels were transferred to iBlot 2 PVDF Regular Stacks (Invitrogen IB24001) using the iBlot P0 protocol. Blots were fixed in 4% paraformaldehyde in TBS, blocked in I-Block (ThermoFisher Scientific T2015) in PBS-T (PBS containing 0.1% tween-20), and left in primary antibody overnight at 4 °C on a shaker. Primary antibody (GAPDH: AB9485 [1:5000]; syn1: BD610787 [1:1000]; DJ-1: AB76008 [1:5000]) was diluted in I-Block in PBS-T. The following day, blots were washed three times in PBS-T for approximately 10 min each wash. Blots were put in the appropriate secondary antibody diluted 1:5000 in I-Block in PBS-T for 45 min at RT on a shaker. Blots were washed again in PBS-T for 10 min for a total of three washes. Blots were developed using ECL reagent. Bands were quantified using LI-COR Image Studio Lite software. The background for this quantification was set to “median” with a border width of three along all the borders.

### Patient-derived αS triplication and corrected neurons (AST23/II8B)

Patient-derived αS triplication and corrected lines (one patient line and one corrected line) were obtained through EBISC and thanks to the Kunath Lab of the University of Edinburgh^95,96^. The corresponding neurogenin-expressing lines were made with the assistance of the BWH iPSC NeuroHub. Lines were maintained as feeder-free cells in defined, serum-free media (mTeSR, Stem Cell Technologies). To generate NGN2-inducible iPSC lines, virus was produced as described previously^97^ with FUW-TetO-Ngn2-P2A-Puromycin (Addgene plasmid #52047) and FUW-M2rtTA (Addgene plasmid #20342). The iPSC line was transduced with each virus at an MOI of 30 and expanded as feeder-free cells in mTeSR. Neural induction was achieved with minor modifications to previous protocols^98^ and as per the method outlined in ref. ^23^.

#### Culture and treatment—human differentiated neurons

NBM complete media was prepared by adding 20% dextrose, GlutaMAX (Gibco 35050), and MEM NEAA (Invitrogen 11140-050) to pure NBM (Gibco 21103) to yield final concentrations of 0.3%, 2 mM, and 1×, respectively. The solution was sterile filtered and stored at 4 °C.

BDNF, CNTF, and GDNF (Peprotech 450-02, 450-13, 450-10, respectively) were dissolved in 0.1% BSA in PBS to make a 10 µg/mL solution. Y-27632 ROCK inhibitor (STEMCELL Technologies 72304) was dissolved in DMSO to make a 10 mM solution. Doxycycline hyclate (Sigma D9891) was prepared as a 20 mg/mL in sterile water. B27 (Life Technologies A11138-03), puromycin (Life Technologies A11138-03), and matrigel (Corning 354234) were used at the manufacturer’s concentrations. Aliquots were stored at −20 °C.

Thawing Media (NBM complete media containing 10 µM Y-27632 ROCK inhibitor), Plating Media (NBM complete media containing 10 ng/mL BDNF, 10 ng/mL CNTF, 10 ng/mL GDNF, 10 µM Y-27632 ROCK inhibitor, 1× B27, 5 µg/mL puromycin, and 2 µg/mL doxycycline hyclate), D7 Media (NBM complete media containing 10 ng/mL BDNF, 10 ng/mL CNTF, 10 ng/mL GDNF, 1× B27, 5 µg/mL puromycin, and 2 µg/mL doxycycline hyclate), and Maintenance Media (NBM complete media containing 10 ng/mL BDNF, 10 ng/mL CNTF, 10 ng/mL GDNF, and 1× B27) were prepared and equilibrated to 37 °C immediately before use.

A day before plating, plates were coated with Matrigel Matrix Basement Membrane (Corning 354234) at 34.8 µg/cm^2^. Coated plates were kept in a 37 °C incubator overnight. The matrigel was aspirated from the wells immediately before plating.

The following day, D4 iN cells were thawed in Thawing Media and plated in Plating Media. A half media change was performed on D7 using D7 Media. A second half media change was performed on D9 using Maintenance Media into which drug additions were incorporated. A third half media change was performed on D21 using Maintenance Media, incorporating additional drug additions. Cells were harvested for various readouts at approximately D25.

#### LDH—human differentiated neurons

A CytoTox 96^®^ Non-Radioactive Cytotoxicity Assay (Promega G1781) was performed according to the manufacturer’s instructions. Briefly, Substrate Mix was dissolved in 12 mL Assay Buffer. A total of 30 μL of cell supernatant was transferred to a 96-well assay plate (Corning Costar 3603) and combined with 30 μL of substrate solution. The plate was incubated at RT for 30 min in the dark. After that time, 30 μL of Stop Solution was added to each well. The absorbance was measured at 490 nm using a BMG LABTECH CLARIOstar plate reader. Samples were assayed in duplicate.

#### Crosslinking—human differentiated neurons

Cells were plated at a density of 200,000 cells per well in a 12-well plate and maintained as described above. On approximately D25, cells were harvested for crosslinking. Briefly, cells were transferred to Eppendorfs in DPBS and pelleted by spinning at 500 × g for 5 min at RT. Pellets were resuspended in 0.25 mM DSG in DBPS with PPI and crosslinked for 30 min at 37 °C with gentle agitation. The remainder of the protocol is as per “Crosslinking—neuroblastoma cells” above.

#### Phosphorylation assays—human differentiated neurons

Cells were plated at a density of 200,000 cells per well in a 24-well plate and maintained as described above. On approximately D25, cells were harvested for the following phosphorylation assay. Media was aspirated from wells and cells were transferred to Eppendorfs in ice-cold DPBS. Cells were spun at 500 × g for 5 min. Pellets were resuspended in 40 μL of modified RIPA lysis buffer (50 mM Tris pH 7.4, 1% Triton-X, 0.1% sodium deoxycholate, 0.1% SDS, 150 mM NaCl, 1 mM EDTA) containing PPI. Cells were lysed on ice for 10 min and then spun at approximately 20,000 × g for 10 min. The remainder of the protocol is as per “Phosphorylation assays—neuroblastoma cells” above.

#### Unfolded protein response—human differentiated neurons

This assay was completed as per the phosphorylation assay above with the modification that gels were transferred to PVDF membranes using the iBlot P0 protocol, fixed in 4% paraformaldehyde in TBS for 5 min, blocked in I-Block in TBS-T for 30 min, and left in primary antibody overnight at 4 °C on a shaker. Primary antibody (Ire1α: NB100-2323 [1:1000]; GAPDH AB9485 [1:5000]) was diluted in I-Block in TBS-T. The following day, blots were washed three times in TBS-T for approximately 10 min each wash. Blots were put in 1:5000 of the appropriate secondary antibody diluted in I-Block in TBS-T for 45 min at RT on a shaker. Blots were washed again in TBS-T for 10 min for a total of three washes before developing using ECL reagent.

#### αS sequential extraction—human differentiated neurons (modified from ref. ^75^)

Cell pellets were harvested for the corrected line, patient-derived αS triplication neurons, and patient-derived neurons treated with LIPEi. Pellets were dissolved pellet in TBS + 1% Triton-X (+Protease Inhibitor) and incubated on ice, 15 min. Samples were spun in an ultracentrifuge at 50,000 rpm, 20 min, 4 °C. The supernatant was removed (the Tx fraction) and the pellet dissolved in RIPA buffer (TBS + 1%NP-40 + 0.5% SDS), incubated on ice, 10 min, spun in an ultracentrifuge at 50,000 rpm, 20 min, 4 °C. The supernatant was removed (the RIPA fraction) and the pellet dissolved in UREA/SDS (8 M + 5% SDS) (UREA/SDS fraction).

#### αS sequential extraction (cytosolic αS:membrane αS)—human differentiated neurons

Neurons were subjected to in situ (on-plate) extraction of (i) cytosolic proteins; (ii) membrane-bound proteins per^77^. Briefly, Neurons were washed with HBSS (37 °C). Cytosol buffer containing 1200 μg/mL digitonin was added to the cells. Plates were incubated at 37 °C, 15 min. The resulting cytosolic protein fraction was collected. Membrane buffer was added to the cells and incubated at 37 °C, 15 min. The resulting membrane fraction was collected.

#### ICC—human differentiated neurons

ICC and microscopy: cultures were fixed with 4% paraformaldehyde, followed by membrane permeabilization with 0.2% Triton-X-100 (Sigma) and 2% donkey serum (Jackson ImmunoResearch Laboratories). They were subsequently stained with primary and secondary antibodies (see Antibodies below). Imaging was performed using a Zeiss LSM710 confocal microscope and images were acquired using ZEN black software. Software was used to pseudo-color images and add scale bars.

Antibodies and stains: immunostaining was performed with the following antibodies: Tau (Dako A0024, 1:200), Syn (BD, 610787), and LipidSpot™ 610 Lipid Droplet Stain (Biotium 70069, 1:1000). Secondaries were from Jackson ImmunoResearch Laboratories: anti-rabbit cy2/cy3, anti-mouse cy2/cy3. DAPI (Invitrogen D1306, 1:1000).

Analysis-measurement of corrected total cell body cluster fluorescence: using confocal images of ICC-stained iPSC-derived human neurons, total fluorescence intensities for αS staining in cell body clusters were measured with “ImageJ”. All cell body clusters with clear DAPI labeled nuclei were traced by free-hand lines based on TAU staining and measured for the area and integrated fluorescence density for αS. Mean background fluorescence intensity was measured from a non-fluorescent region of the same image to account for non-specific signals. The corrected total fluorescence of each cell body cluster normalized to the area of cell body cluster and the background fluorescence was then calculated using the formula: Integrated Density – (Area of Cell Body Cluster × Mean Background Fluorescence).

Assessment of colocalization of αS with LDs: degree of colocalization between αS and LD staining was quantified using the “Just Another Colocalization Plugin (JACoP)” program in “ImageJ”. The multi-channel confocal images of ICC-stained neurons were split into single-channel images. On the JACoP plugin, Mander’s coefficients (M1 and M2) (MOC) were measured by selecting the image with αS staining as “Image A” and the same image with LD staining as “Image B”. The images were manually thresholded with a value of 90 for the αS channel and 30 for the LD channel.

### *C. elegans* strains and maintenance

The *C. elegans* strains UA44 (*baIn11*[P_*dat-1*_::α-syn, P_*dat-1*_::GFP]) expressing αS and GFP in DA neurons and BY250 (*vtIs7*[P_*dat-1*_::GFP]) that expresses GFP only were used in this study^99,100^. Nematodes were maintained using standard procedures^101^.

#### RNA interference (RNAi) bacterial growth conditions

HT115 RNAi bacteria containing L4440 feeding vector (either an empty feeding vector or containing *hosl-1*) were grown on LB plates containing 12.5 μg/mL tetracycline and 100 μg/mL ampicillin for 16 h at 37 °C. Single colonies were transferred to LB broth containing 100 μg/mL ampicillin for 16 h at 37 °C shaking. RNAi bacteria in liquid culture were then seeded onto nematode growth medium plates, which also contained 1 mM IPTG and 100 μg/mL ampicillin, and allowed to dry. Once dried, plates were moved to 20 °C for 18 h to allow for optimal growth at a set temperature. Adult worms were placed onto RNAi plates with the specified feeding vector and allowed to lay eggs for 3 h to allow for synchronization. Adults were removed and progeny were allowed to grow up. Starting at day 3 of adulthood, worms were transferred every day to new RNAi plates until day 8, when dopaminergic neurons were analyzed for neurodegeneration. For experiments in which nematodes were grown for two generations on RNAi, the same method as previously mentioned was used, however, when the F1 progeny reached day 4, these gravid adults were used for a synchronized egg lay onto new RNAi plates and the F2 progeny were analyzed at day 8 of adulthood for dopaminergic neurodegeneration.

#### *C. elegans* dopaminergic neurodegeneration analysis

UA44 and/or BY250 worms were exposed to either EV or *hosl-1* dsRNA HT115 bacteria prior to neuronal analysis. Worms were synchronized and grown at 20 °C and the F1 or F2 generations were analyzed on day 8 post hatching for α-syn-induced dopaminergic neurodegeneration. *C. elegans* dopaminergic neurons were assessed for degeneration as previously described^102^. Briefly, using a Nikon E800 fluorescent microscope, on the day of analysis, 30 adult hermaphrodite worms were immobilized in 10 mM levamisole resuspended in S-basal medium on glass coverslips and placed onto 2% agarose pads on microscope slides. The six anterior dopaminergic neurons, four cephalic, and two anterior deirid neurons of each worm were examined for deformities such as dendrite blebbing, dendrite loss, cell body rounding, and missing cell bodies. A worm with at least one degenerative change was classified as exhibiting neurodegeneration^102^. Nematodes were scored in triplicate (30 worms/replicate for 3 replicates) for a total of 90 adult worms analyzed per treatment. For statistical analysis, an unpaired Student’s *t*-test (*p* < 0.05) was employed using GraphPad Prism (version 8).

#### *C. elegans* RNA extraction and reverse transcription qPCR

qPCR reactions were performed using IQSYBR Green Supermix (Bio-Rad, Hercules, CA) with the CFX96 Real-Time System (Bio-Rad) as described previously^103^. Worms were washed three times in RNase free .5X M9 followed by a single wash in RNase free water. Total RNA was isolated from 100 to 120 adult worms (BY250 or UA44) from each independent sample using TRI reagent (Molecular Research Center) on day 8 of adulthood, following either one generation (UA44) or two generations (UA44 and BY250) of hosl-1 or EV RNAi exposure. Following DNase treatment (Promega, Madison, WI), 1 μg of RNA was used to make complementary DNA (cDNA), which was synthesized with iScript Reverse Transcription Supermix for qRT-PCR (Bio-Rad, Hercules, CA, USA). PCR efficiency was calculated from standard curves that were generated using serial dilutions of cDNA of all samples. All targeted genes were measured in triplicate. Amplification was not detected in non-template and non-reverse transcriptase controls. Each reaction contained: 7.5 μL of the IQSYBR Green Supermix, 200 nM of forward and reverse primers, and 0.3 μL cDNA, to a final volume of 15 μL. Expression levels were normalized to three reference genes (cdc-42, ama-1, and pmp-3) and were calculated using qBasePLUS version 2.6 (Biogazelle, Gent, Belgium) for determining reference target stability. Three technical replicates were used for each sample. Each primer pair was confirmed for at least 90–110% efficiency in a standard curve on BY250 cDNA. First-generation RNAi UA44, *N* = 4; *n* = 3; second-generation RNAi BY250, *N* = 4; *n* = 3; second-generation RNAi UA44, for hosl-1 *N* = 4; *n* = 3 and EV *N* = 2; *n* = 3. The following primer sequences were used for the experiments:

hosl-1 Forward GCCAGTTGTTCAGACAGC

hosl-1 Reverse GTAGAAGCGTGGTGTCG

pmp-3 Forward GTT CCC GTG TTC ATC ACT CAT

pmp-3 Reverse ACA CCG TCG AGA AGC TGT AG

cdc-42 Forward CCG AGA AAA ATG GGT GCC TG

cdc-42 Reverse TTC TCG AGC ATT CCT GGA TCA T

ama-1 Forward TCC TAC GAT GTA TCG AGG CAA

ama-1 Reverse CTC CCT CCG GTG TAA TAA TGA

### αS E46K-expressing neurons

The established 2132 iPSC line from a previously clinically characterized healthy individual^104^ was transduced with TetO-Ngn2-Puro as described^105^ to establish “NR” (neurogenin-2 + rtTA) iPSCs. NR iPSCs were then transduced with pLVX-EF1a/αS-IRES-mCherry lentiviral plasmids for E46K (one line)^83^. Neurons were grown on poly-L-ornithine/laminin pre-coated plates (Biocoat).

#### Culture and treatment—αS E46K-expressing neurons

BDNF, CNTF, and GDNF (Peprotech 450-02, 450-13, 450-10, respectively) were dissolved in 0.1% BSA in PBS to make a 10 µg/mL solution. Y-27632 ROCK inhibitor (STEMCELL Technologies 72304) was dissolved in DMSO to make a 10 mM solution. Doxycycline hyclate (Sigma D9891) was prepared as a 20 mg/mL in sterile water. B27 Plus Supplement (Gibco A3582801), puromycin (Life Technologies A11138-03), and laminin mouse protein (Gibco 23017015) were used as prepared by the manufacturer.

Complete neurobasal plus media (NBM+) was prepared by adding 20% w/v dextrose, GlutaMAX (Gibco 35050), and MEM NEAA (Invitrogen 11140-050) to pure NBM+ (Gibco A3582901) to yield final concentrations of 0.3%, 2 mM, and 1×, respectively. The solution was sterile filtered and stored at 4 °C.

Thawing Media (complete NBM+ media containing 10 µM Y-27632 ROCK inhibitor), Plating Media (complete NBM+ media containing 10 ng/mL BDNF, 10 ng/mL CNTF, 10 ng/mL GDNF, 10 µM Y-27632 ROCK inhibitor, 1× B27 Plus Supplement, 10 µg/mL puromycin, 2 µg/mL doxycycline hyclate, and 0.5–2.0 µg/mL laminin), and Maintenance Media (complete NBM+ media, 10 ng/mL BDNF, 10 ng/mL CNTF, 10 ng/mL GDNF, 1× B27 Plus Supplement, 10 µg/mL puromycin, 2 µg/mL doxycycline hyclate) were prepared and equilibrated to 37 °C in a bead bath immediately before use.

D4 iN cells were thawed in Thawing Media and plated in Plating Media at a density of 250,000 cells per well in 24-well poly-L-ornithine/laminin plates (Corning 354659). Cells received half media changes on D5, D8, D10, D19, and D22 using Maintenance Media. On D10 and D22, cells were also treated with drug. Cells were harvested on approximately D25.

#### Phosphorylation assays—αS E46K-expressing neurons

On approximately D25, cells were harvested according to “Phosphorylation assays—human differentiated neurons” described above.

### FA sample preparation, profiling, and analysis (OmegaQuant)

Cell culture FA composition was analyzed at OmegaQuant by GC with flame ionization detection. Cells were harvested and resuspended in 80% methanol. The methanol and pellet samples were transferred into their own respective screw-cap glass vial and dried down using an Organomation Associates Inc. nitrogen evaporator. After drying the methanol and pellet samples, 14% boron trifluoride–methanol (Sigma-Aldrich, St. Louis, MO) and hexane (EMD Millipore Chemicals, USA) were added. The vials were capped, vortexed, and centrifuged to separate the layers.

For the free fatty acid analysis, a portion of the hexane layer was taken and transferred to a new screw-cap glass vial. An amount of 4% boron trifluoride–methanol (Sigma-Aldrich, St. Louis, MO) was then added to each vial. The vials were capped, vortexed, and placed on a Hoefer Pharmacia Biotech Inc. platform shaker in a 4 °C refrigerator for 150 min. Immediately after, HPLC grade water was added. The vials were recapped, vortexed, and centrifuged to separate layers. An aliquot of the hexane layer was transferred to a GC vial to be injected onto the GC-FID.

For the phospholipid analysis, a portion of the 14% boron trifluoride layer was taken and transferred to a new screw-cap glass vial. The vial was capped, vortexed, and heated in a hot bath at 100 °C for 10 min. After cooling, hexane (EMD Millipore Chemicals, USA) and HPLC grade water were added sequentially. The vials were recapped, vortexed, and centrifuged to separate layers. An aliquot of the hexane layer was transferred to a GC vial for injection onto the GC-FID. GC-FID was carried out using a GC2010 Gas Chromatograph (Shimadzu Corporation, Columbia, MD) equipped with a Supelco SP2560 fused silica capillary column (100 m × 0.25 mm internal diameter × 0.2 μm film thickness; Supelco, Bellefonte, PA).

Fatty acids were identified by comparison with a standard mixture of fatty acids characteristic of RBC (GLC OQ-A, NuCheck Prep, Elysian, MN), which was also used to determine individual fatty acid calibration curves.

### Lipid sample preparation, profiling, and analysis (Lipotype)

Mass spectrometry-based lipid analysis was performed by Lipotype GmbH (Dresden, Germany) as described^106^. Lipids were extracted using a two-step chloroform/methanol procedure^107^. Samples were spiked with internal lipid standard mixture containing: cardiolipin 14:0/14:0/14:0/14:0 (CL), ceramide 18:1;2/17:0 (Cer), diacylglycerol 17:0/17:0 (DAG), hexosylceramide 18:1;2/12:0 (HexCer), lyso-phosphatidate 17:0 (LPA), lyso-phosphatidylcholine 12:0 (LPC), lyso-phosphatidylethanolamine 17:1 (LPE), lyso-phosphatidylglycerol 17:1 (LPG), lyso-phosphatidylinositol 17:1 (LPI), lyso-phosphatidylserine 17:1 (LPS), phosphatidate 17:0/17:0 (PA), phosphatidylcholine 17:0/17:0 (PC), phosphatidylethanolamine 17:0/17:0 (PE), phosphatidylglycerol 17:0/17:0 (PG), phosphatidylinositol 16:0/16:0 (PI), phosphatidylserine 17:0/17:0 (PS), cholesterol ester 20:0 (CE), sphingomyelin 18:1;2/12:0;0 (SM) and triacylglycerol 17:0/17:0/17:0 (TAG). After extraction, the organic phase was transferred to an infusion plate and dried in a speed vacuum concentrator. First step dry extract was resuspended in 7.5 mM ammonium acetate in chloroform/methanol/propanol (1:2:4,V:V:V) and second step dry extract in 33% ethanol solution of methylamine in chloroform/methanol (0.003:5:1; V:V:V). All liquid handling steps were performed using Hamilton Robotics STARlet robotic platform with the Anti Droplet Control feature for organic solvents pipetting.

Samples were analyzed by direct infusion on a QExactive mass spectrometer (ThermoScientific) equipped with a TriVersa NanoMate ion source (Advion Biosciences). Samples were analyzed in both positive and negative ion modes with a resolution of Rm/z = 200 = 280,000 for MS and Rm/z = 200 = 17,500 for MSMS experiments, in a single acquisition. MSMS was triggered by an inclusion list encompassing corresponding MS mass ranges scanned in 1 Da increments^108^. Both MS and MSMS data were combined to monitor CE, DAG, and TAG ions as ammonium adducts; PC, PC O-, as acetate adducts; and CL, PA, PE, PE O-, PG, PI, and PS as deprotonated anions. MS only was used to monitor LPA, LPE, LPE O-, LPI, and LPS as deprotonated anions; Cer, HexCer, SM, LPC, and LPC O- as acetate adducts.

Data were analyzed with in-house developed lipid identification software based on LipidXplorer^109,110^. Data post-processing and normalization were performed using an in-house developed data management system. Only lipid identifications with a signal-to-noise ratio >5, and a signal intensity 5-fold higher than in corresponding blank samples were considered for further data analysis.

### FA statistical analysis and heatmap representations

Raw FA data were calculated based on the area under the curve and a three-level calibration curve was used to determine values based on a ratio of a given fatty acid to an internal standard. Missing/zero values were imputed with the half of the minimum value for the FA across samples [https://academic.oup.com/bioinformatics/article/34/9/1555/4764003]. To account for the data’s skewness and heteroscedasticity, raw abundance FA data were normalized by log2-transformation and center scaling [https://www.ncbi.nlm.nih.gov/pmc/articles/PMC1534033/] and heatmaps drawn using the /pheatmap/ [https://cran.r-project.org/web/packages/pheatmap/index.html] package in R. An average abundance heatmap for each treatment group is also provided to highlight the more abundant FAs. The data were examined via principal component analysis and hierarchical clustering to identify outliers and potential confounding factors. Finally, differentially abundant FAs were identified using /Limma/ [http://bioconductor.org/packages/release/bioc/html/limma.html] via /ezlimma/ [https://github.com/jdreyf/ezlimma] package in R, for the pairwise comparisons between treatment groups. Information on FA identity, including chain length and saturation for Figs. 1C, 2A, and 4F and Supplementary Figs 1D, G, 2F, G, 3A, B, E, and 4F, I, is outlined in Supplementary Table 2.

## Supplementary information

Supplementary Materials

## Data Availability

All data generated or analyzed during this study are included in this published article (and its Supplementary information files).
