# Supplementary material for: Lipase regulation of cellular fatty acid homeostasis as a Parkinson’s disease therapeutic strategy
Source: NPJ Parkinsons Dis. 2022 Jun 9;8:74. doi: 10.1038/s41531-022-00335-6 (PMC9184586; doi:10.1038/s41531-022-00335-6)
Supplement: Supplementary file 1 — Supplementary Materials [file 41531_2022_335_MOESM1_ESM.pdf]

Supplementary Material

Lipase Regulation of Cellular Fatty Acid Homeostasis

as a Parkinson’s Disease Therapeutic Strategy

Saranna Fanning[1*], Haley Cirka[1], Jennifer L Thies[2], Jooyoung Jeong[1], Sarah M Niemi[1], Joon Yoon[5], Gary P H Ho[1], Julian A Pacheco[3], Ulf Dettmer[1], Lei Liu[1], Clary B Clish[3], Kevin J Hodgetts[4], John N Hutchinson[5], Christina R Muratore[1], Guy A Caldwell[2], Kim A Caldwell[2], Dennis Selkoe[1*].

1. Ann Romney Center for Neurologic Diseases, Department of Neurology, Brigham and Women's Hospital and Harvard Medical School, Boston, MA 02115, USA.
2. Department of Biological Sciences, The University of Alabama, Tuscaloosa, AL 35487, USA.
3. Broad Institute of MIT and Harvard, Cambridge, MA 02142, USA.
4. Laboratory for Drug Discovery in Neuroscience, Department of Neurology, Brigham and Women's Hospital, Boston, MA 02115, USA.
5. Department of Biostatistics, The Harvard Chan School of Public Health, Boston, MA 02115, USA.

* Corresponding authors: sfanning2@bwh.harvard.edu dselkoe@bwh.harvard.edu

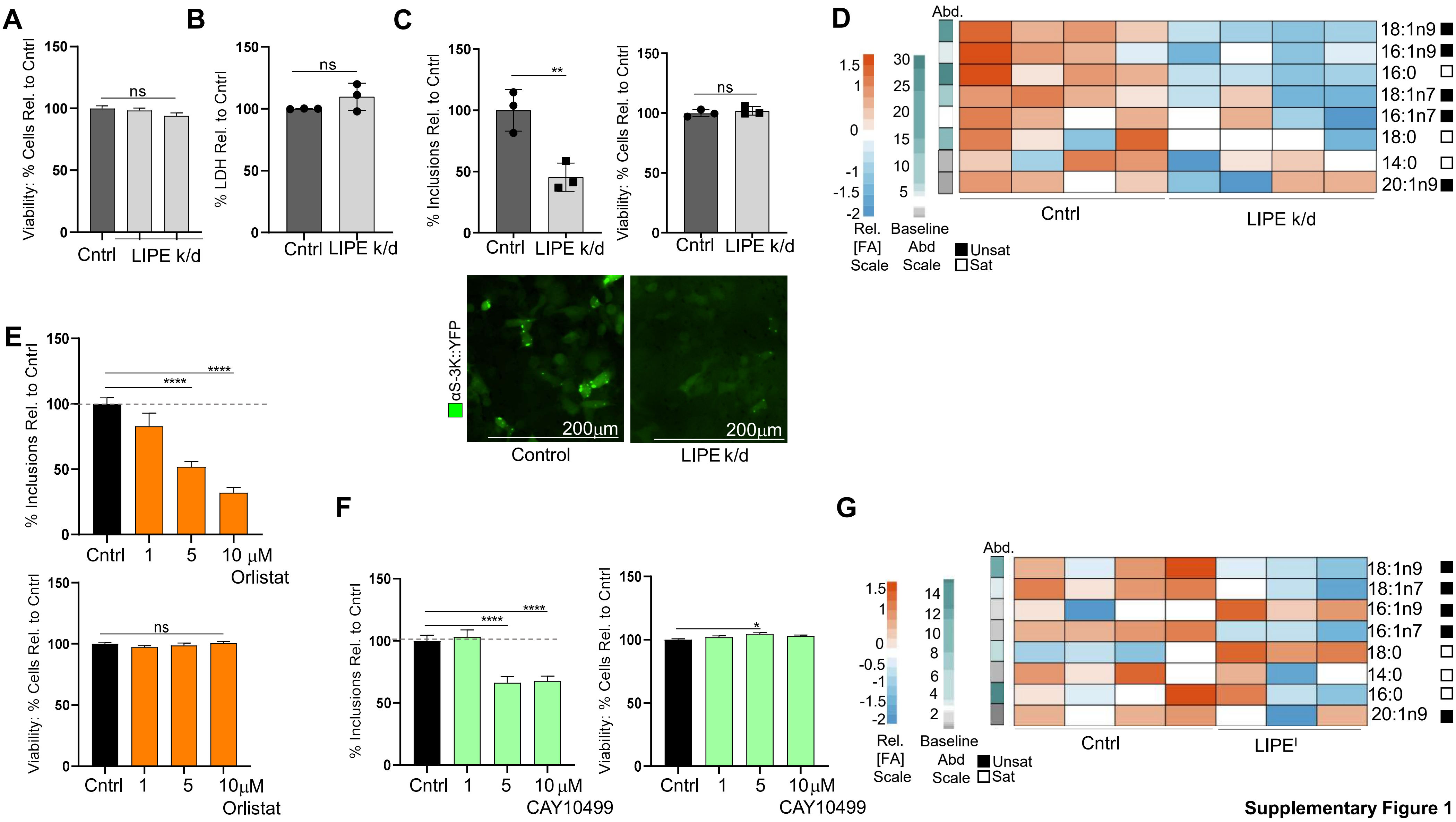

Supplementary Figure 1

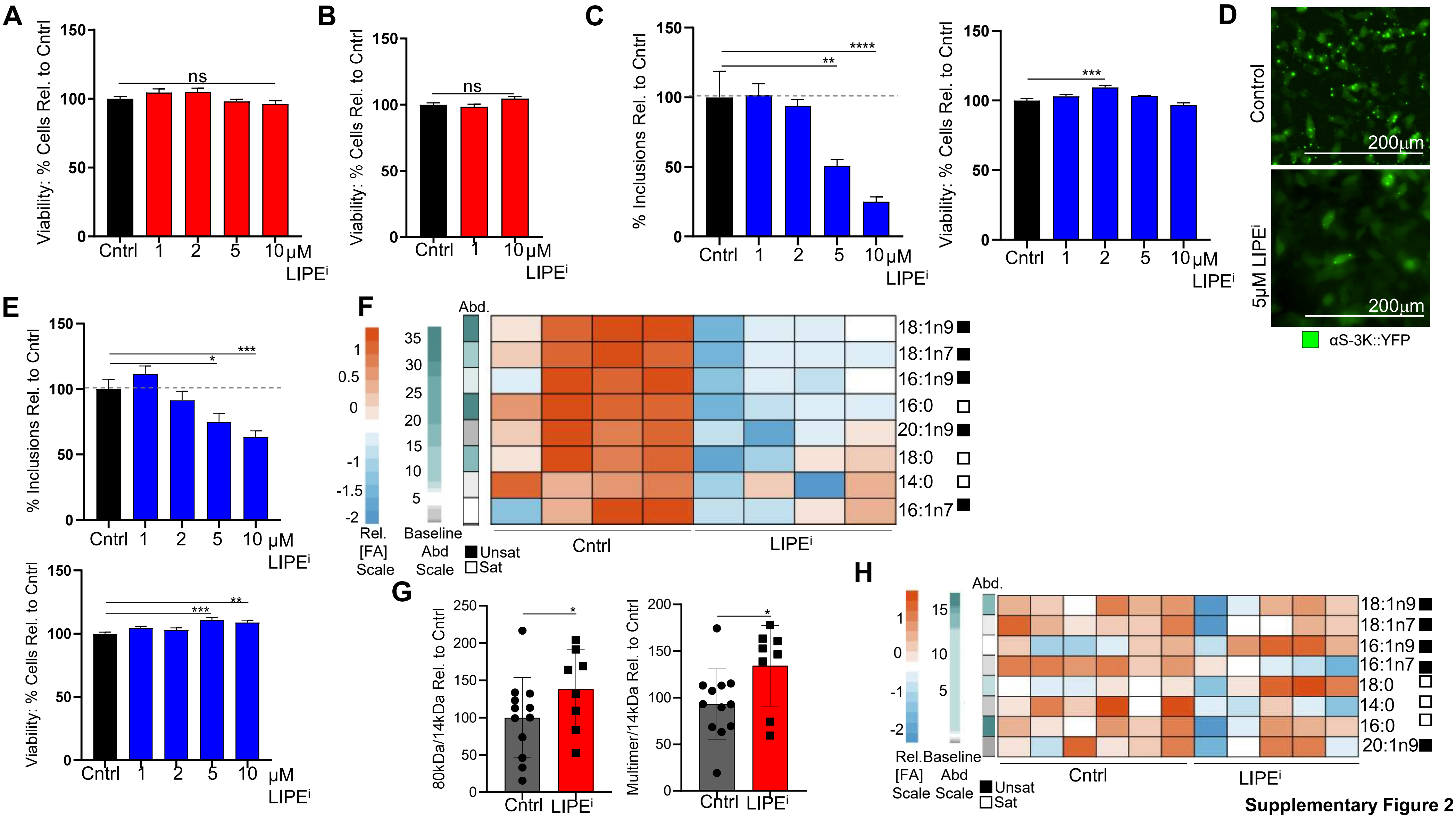

Supplementary Figure 2

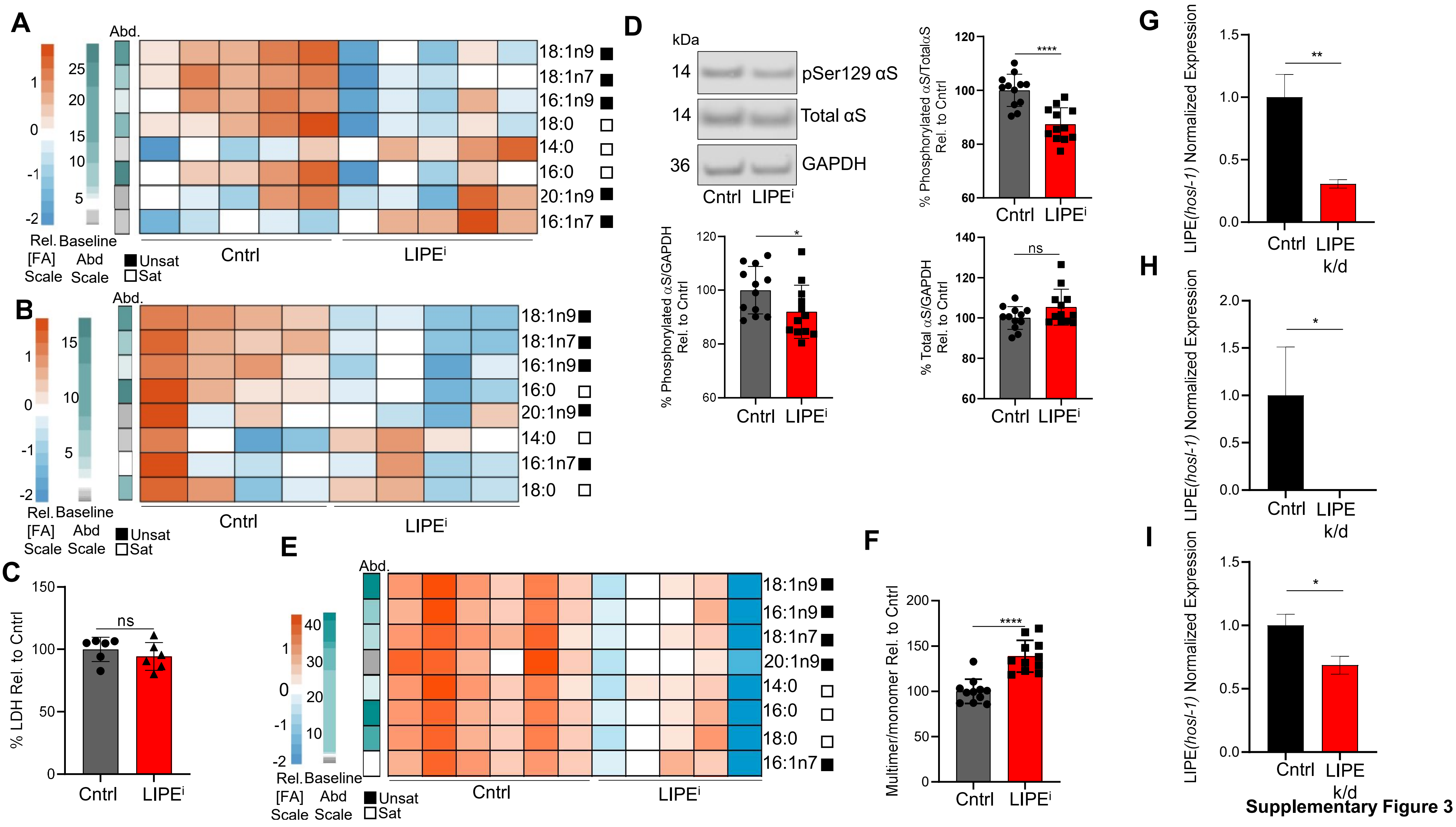

Supplementary Figure 3

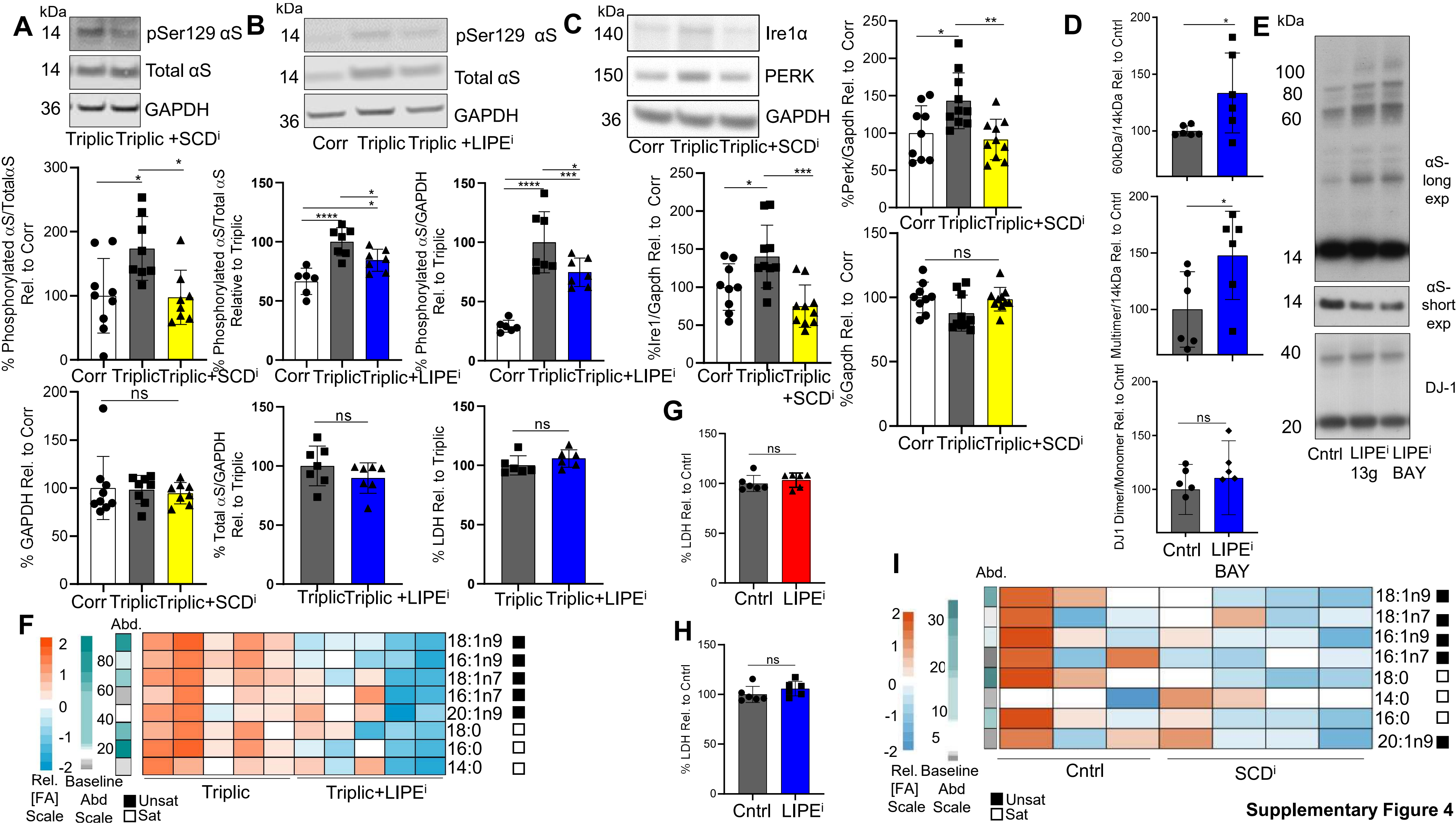

Supplementary Figure 4

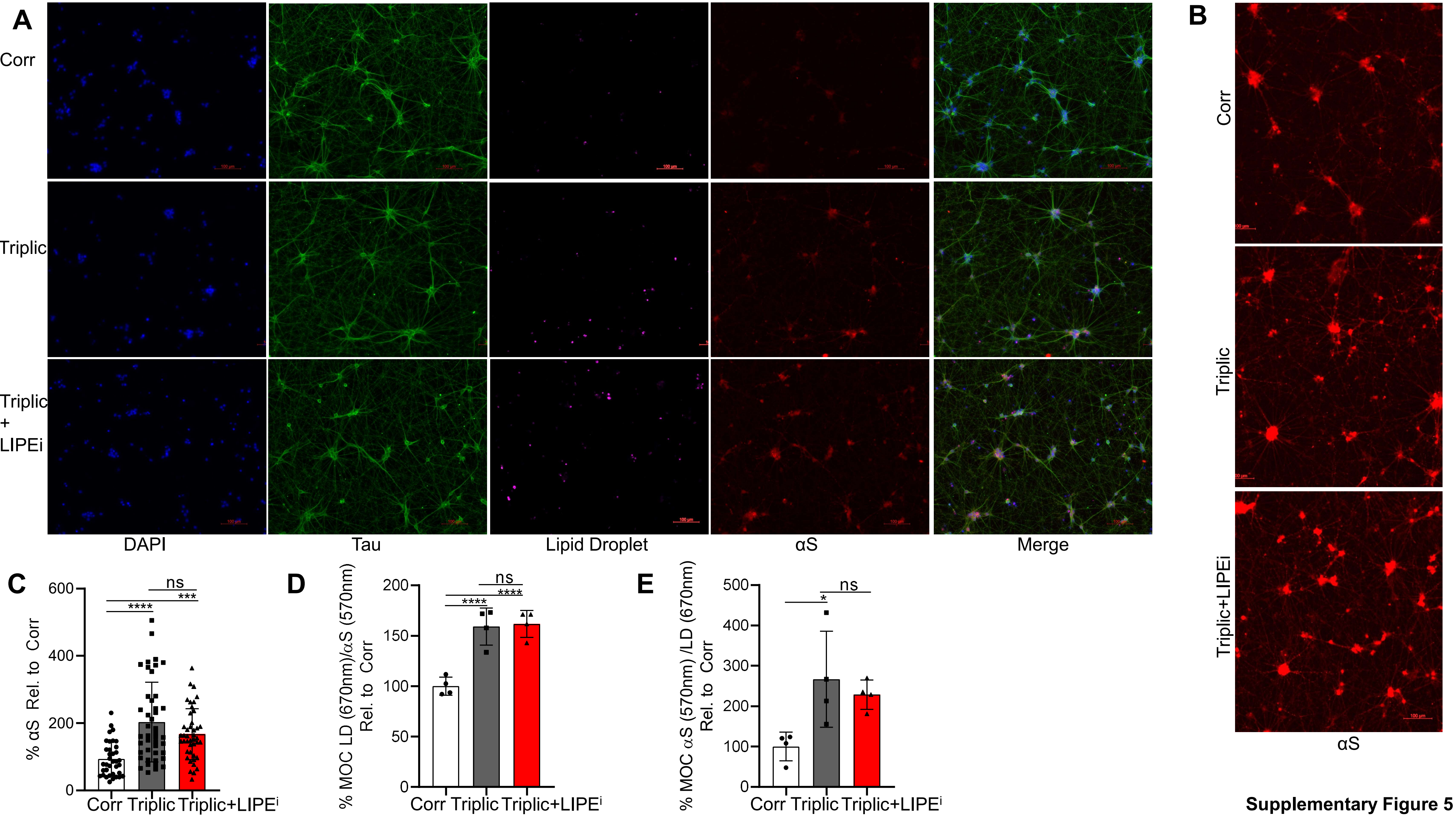

Supplementary Figure 5

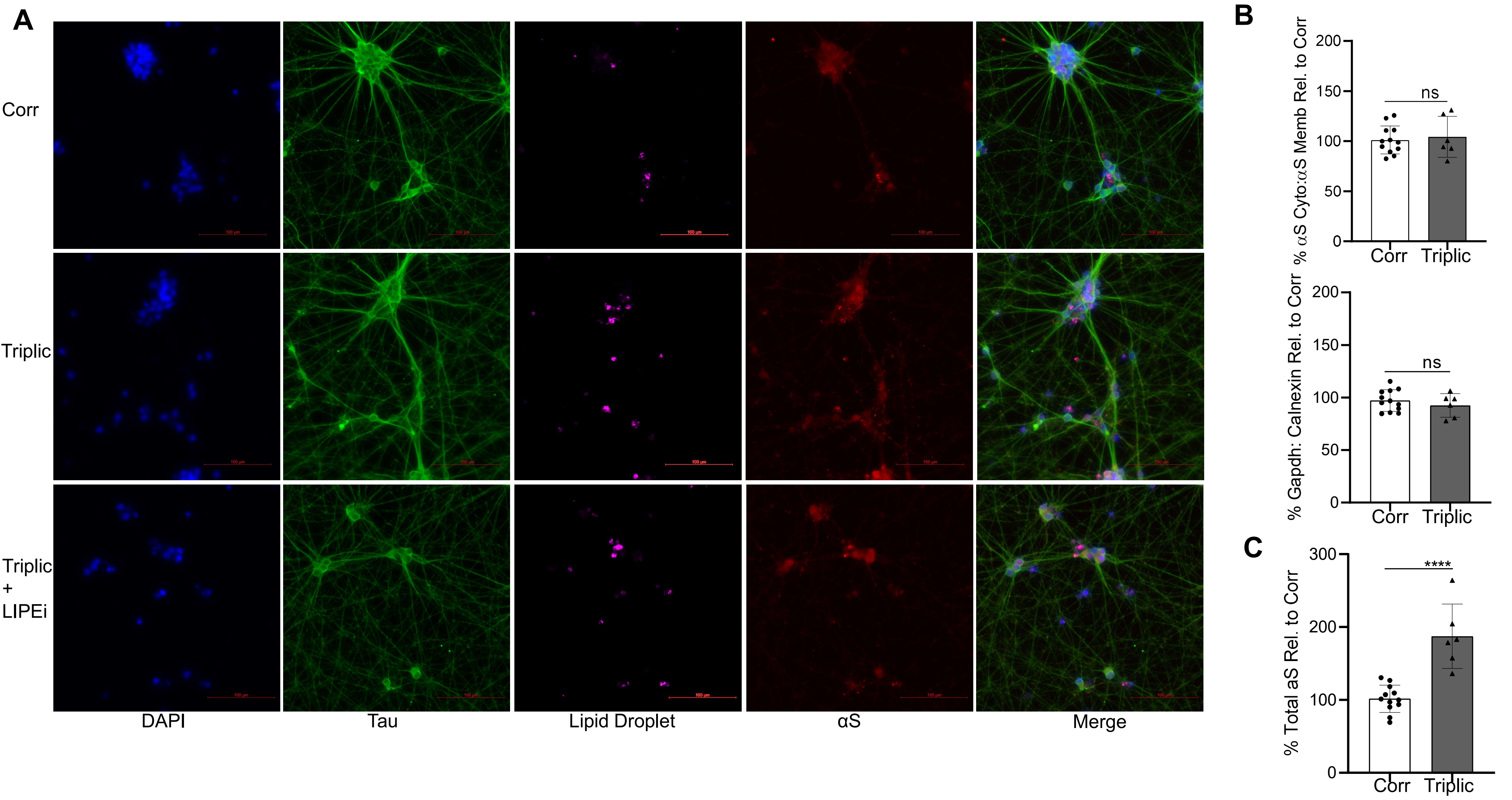

Supplementary Figure 6

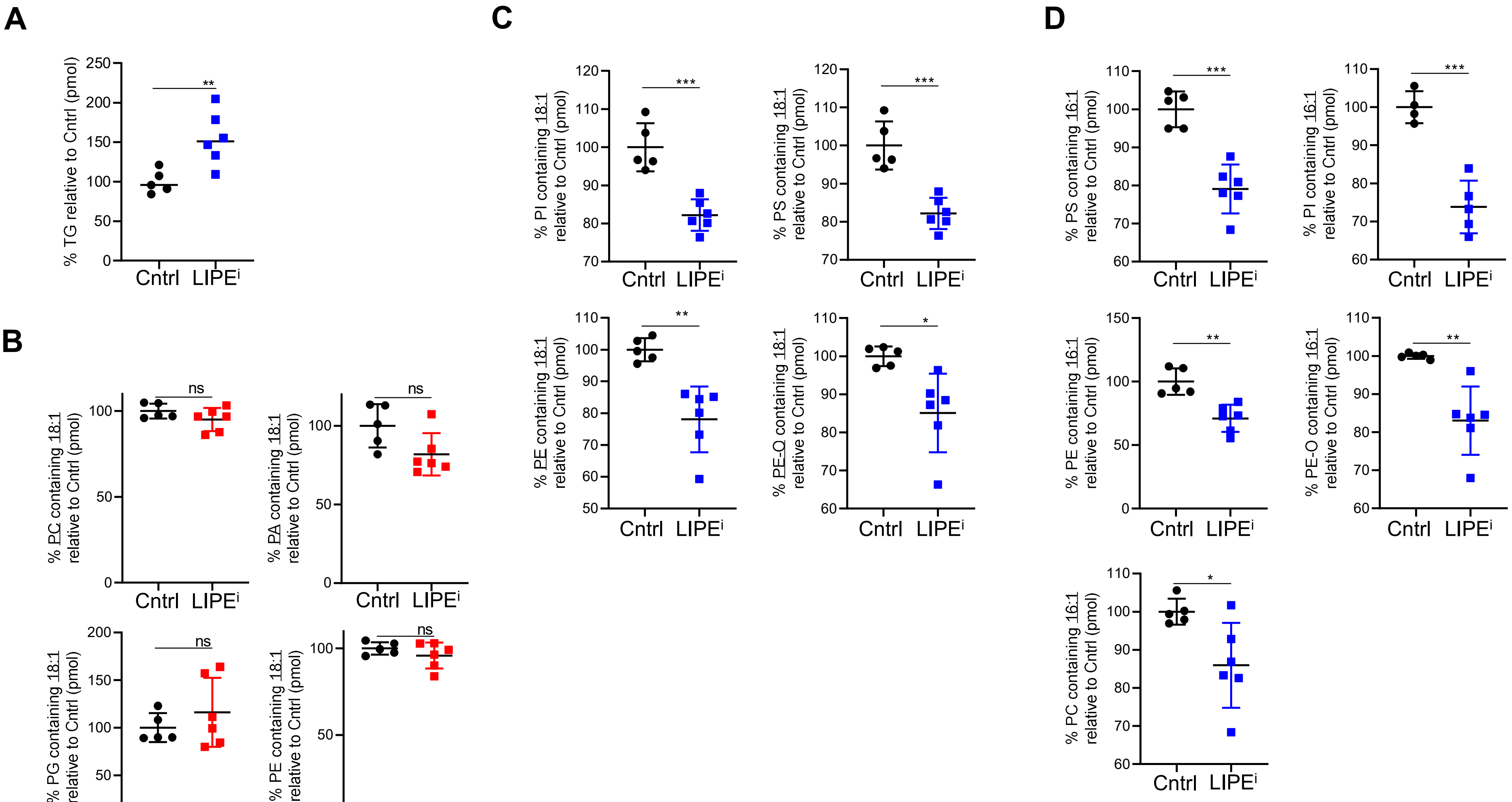

Supplementary Figure 7

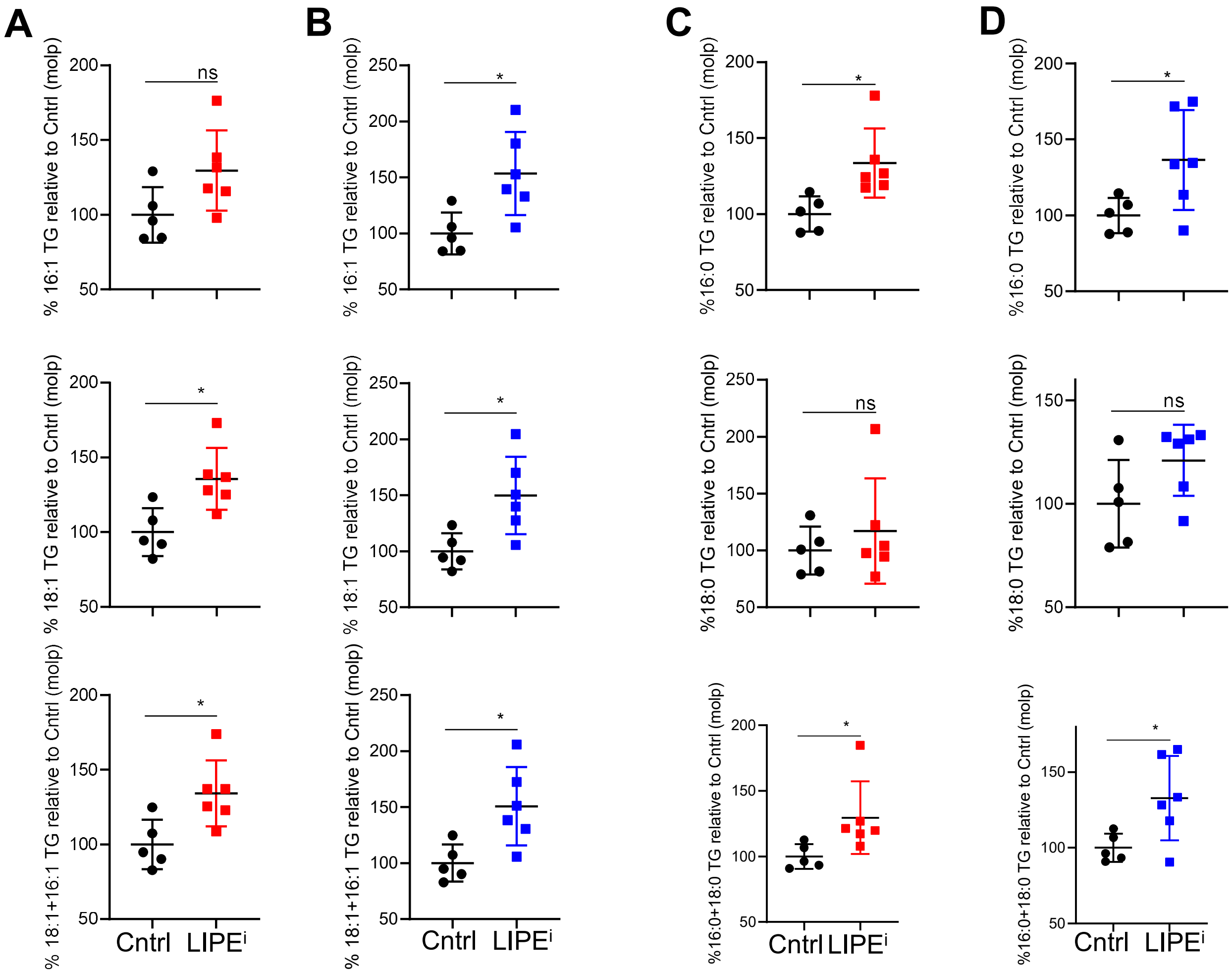

Supplementary Figure 8

SUPPLEMENTARY FIGURE LEGENDS

Supplementary Figure 1: Reducing lipase activity reduces PD relevant phenotypes in an αS 3K model

(A) **Genetic knockdown of LIPE does not impact cell viability.** Cell viability was assayed by mCherry expression data for shRNA LIPE knockdown relative to control (Cntrl) in M17D/αS-3K::YFP cells. Y-axis is viability: % cells relative to the control. Bars represent mean values and error bars represent standard error of mean. Statistical analysis by one-way Anova (Graphpad Prism 8). This viability data relates to Fig 1B. (B) **Genetic knockdown of LIPE does not impact cell viability.** Cell viability was assayed by lactate dehydrogenase (LDH) release using Promega G1780 as per manufacturer's instructions. Y-axis is % LDH relative to the control. Bars represent mean values and error bars represent standard deviation. n=3. (C) **LIPE knockdown decreases αS inclusion formation**. As per Fig. 1B. Bars represent mean values and error bars represent standard error of mean. n=3. Microscopy images show decreased inclusions (green channel) upon LIPE knockdown. Images are representative of at least 8 images shown in Fig. 1B. (D) **Genetic Reduction of LIPE alters phospholipid-incorporated FAs**. Full heatmap associated with Fig. 1C. FA saturated/unsaturated status is indicated by white/black bars to the right of FA species. (E) **Inhibition of lipase activity (Orlistat) decreases αS inclusion formation dose-dependently in a pre-treatment paradigm without altering cell viability**. M17D/αS-3K::YFP cells were incubated for 16 h with doses of Orlistat. αS expression was induced and the number of inclusions were measured after 24 h of induction. Inclusions were expressed as % compared to the control (Cntrl) (n in graph order: 12, 11, 10, 11). Dotted line represents 100 % inclusions in the control. Y-axis is % αS inclusions relative to the control. Bars represent mean and error bars represent standard error of mean. ****$p<0.0001$ by one-way

Anova (Graphpad Prism 8). Cell viability was assayed by mCherry expression data relative to control (Cntrl). Y-axis is viability: % cells relative to the control. Error bars represent standard error of mean. (F) **Inhibition of lipase activity (CAY10499) decreases αS inclusion formation dose-dependently in a pre-treatment paradigm without decreasing cell viability**. M17D/αS-3K::YFP cells were incubated for 16 h with doses of CAY10499. αS expression was induced and the number of inclusions were measured after 24 h of induction. Inclusions were expressed as % compared to the control (Cntrl) (n in graph order: 12, 11, 10, 12). Dotted line represents 100 % inclusions in the control. Y-axis is % αS inclusions relative to the control. Bars represent mean and error bars represent standard error of mean. ****$p<0.0001$ by one-way Anova (Graphpad Prism 8). Cell viability was assayed by mCherry expression data relative to control (Cntrl). Y-axis is viability: % cells relative to the control. Error bars represent standard error of mean. Data was analyzed by one-way Anova (Graphpad Prism 8). **(G) LIPE inhibition (13g at 10μM) decreases monounsaturated FAs.** FA profiles of cells treated as per Fig 1D analyzed by gas chromatography. Samples were harvested for FA analysis and analyzed by gas chromatography. Heat maps and statistical analysis were conducted as per materials & methods section. Baseline abundance (Abd) of each FA species is indicated by a green/gray bar on the left of the heat map. Baseline abundance was calculated based on relative amount of each FA species in the control cells. Red/Blue heatmap coloring is a representation of a given FA species (as per analysis outlined in materials & methods section) FA species are indicated on the right of the heatmap. FA saturated/unsaturated status is indicated by white/black bars to the right of FA species.

Supplementary Figure 2: Reducing lipase activity reduces PD relevant phenotypes

(A) **Pharmacological knockdown of LIPE (LIPEi) 13g does not impact cell viability in a pre-treatment paradigm.** Cell viability was assayed by mCherry expression relative to control (Cntrl). Y-axis is viability: % cells relative to the control. Bars represent mean and error bars represent standard error of mean. This viability data relates to data shown in Fig 1D. Data was analyzed by one-way Anova (Graphpad Prism 8). (B) **Pharmacological knockdown of LIPE (LIPEi) 13g does not impact cell viability in a treatment paradigm.** Cell viability was assayed by mCherry expression relative to control (Cntrl). Y-axis is viability: % cells relative to the control. Bars represent mean and error bars represent standard error of mean. This viability data relates to data shown in Fig 1F. Data was analyzed by one-way Anova (Graphpad Prism 8). (C) **LIPE inhibition (LIPEi) (BAY) decreases αS inclusion formation dose-dependently in a pre-treatment paradigm without decreasing cell viability**. M17D/αS-3K::YFP cells were incubated for 16 h with doses of LIPE inhibitor (BAY). αS expression was induced and the number of inclusions were measured after 24 h of induction. Inclusions were expressed as % compared to the control (Cntrl) (n in graph order: 12, 14, 12, 14, 14). Dotted line represents 100 % inclusions in the control. Y-axis is % αS inclusions relative to the control. Bars represent mean and error bars represent standard error of mean. ** $p<0.001$, ****$p<0.0001$ by one-way Anova (Graphpad Prism 8). Cell viability was assayed by mCherry expression data relative to control (Cntrl). Y-axis is viability: % cells relative to the control. Error bars represent standard error of mean ***$p<0.001$ by one-way Anova (Graphpad Prism 8). (D) **LIPE inhibition (LIPEi) (BAY) decreases αS inclusion formation in a pre-treatment paradigm**. Microscopy images showing decreased inclusions (green channel) upon treatment with 5 μM BAY. Images are representative of at least 8 images shown in (C). **(E) LIPE inhibition (LIPEi) (BAY) decreases αS inclusion formation in a treatment paradigm**. αS was induced for 20 h and then cells were

treated with doses of BAY for 16 h. Inclusions were analyzed and expressed as % relative to control (Cntrl). Dotted line represents 100 % inclusions in the control. Y-axis is % αS inclusions relative to the control. Bars represent mean and error bars represent standard error of mean. *$p<0.05$, ***$p<0.001$ by one-way Anova (Graphpad Prism 8). (n in graph order: 13, 13, 14, 14, 13). Cell viability was assayed by mCherry expression data relative to control (Cntrl). Y-axis is viability: % cells relative to the control. Error bars represent standard error of mean **$p<0.01$, ***$p<0.001$ by one-way Anova (Graphpad Prism 8). (F) **Reducing LIPE activity using LIPEi (BAY) reduces monounsaturated FAs.** Control and 10 uM BAY-treated samples were harvested for FA analysis and analyzed by gas chromatography. Heat maps and statistical analysis were conducted as per materials & methods section. Baseline abundance (Abd) of each FA species is indicated by a green/gray bar on the left of the heat map. Baseline abundance was calculated based on relative amounts of each FA species in the control cells. Red/Blue heatmap coloring is a representation of a given FA species (as per analysis outlined in materials & methods section) FA species are indicated on the right of the heatmap. FA saturated/unsaturated status is indicated by white/black bars to the right of FA species. (G) **LIPEi increases αS T:M ratio**. Quantification of 80kDa/14kDa and 60+80kDa/14kDa from Fig 1H. * $p<0.05$ by unpaired t-test (Graphpad Prism 8). Bars represent mean and error bars represent standard deviation. Cntrl n=11, LIPEi n=8 . (H) **LIPEi (13g) primarily decreases monounsaturated FAs in αS E46K expressing neurons.** Samples (as per Fig 2B, 2D) were harvested for FA analysis and analyzed by gas chromatography. Heat maps and statistical analysis were conducted as per materials & methods section. Baseline abundance (Abd) of each FA species is indicated by a green/gray bar on the left of the heat map. Baseline abundance was calculated based on the relative amount of each FA species in the control cells. Red/Blue heatmap coloring is a representation of a given FA

species (as per analysis outlined in materials & methods section). FA species are indicated on the right of the heatmap. FA saturated/ unsaturated status is indicated by white/black bars to the right of FA species.

Supplementary Figure 3: Reducing lipase activity reduces PD relevant phenotypes in E46K expressing cells and in *C. elegans*.

(A) **M17D/αS-3KTD Cells Treated with 20 µM 13g LIPE inhibitor have reduced phospholipid-incorporated monounsaturated FAs.** Samples were harvested for FA analysis and analyzed by gas chromatography. Heat maps and statistical analysis were conducted as per materials & methods section. Baseline abundance (Abd) of each FA species is indicated by a green/gray bar on the left of the heat map. Baseline abundance was calculated based on relative amounts of each FA species in the control cells. Red/Blue heatmap coloring is a representation of a given FA species (as per analysis outlined in materials & methods section) FA species are indicated on the right of the heatmap. FA saturated/unsaturated status is indicated by white/black bars to the right of FA species. (B) **M17D/αS-E46KTD Cells Treated with 20 µM 13g LIPE inhibitor have reduced phospholipid-incorporated monounsaturated FAs.** As per (A) but in neural cells expressing the fPD αS E46K mutation. FA saturated/unsaturated status is indicated by white/black bars to the right of FA species. (C) **Cell viability is not impacted by 13g LIPE inhibition**. Cell viability of αS E46K expressing cells treated with 20 µM 13g was assayed by LDH release (Promega, G1780) relative to control (Cntrl) in M17D/αS-E46K cells. Y-axis is viability: % LDH released relative to the control. Bars represent mean values and error bars represent standard deviation (n=6). (D) **LIPE inhibition (LIPEi) reduces pSer129 αS in αS E46K expressing neural cells**. Cell lysates were immunoblotted to detect and quantify pSer129

αS, total αS and GAPDH control. Bar charts quantify differences between control (Cntrl) and LIPE inhibitor (LIPEi) 20 μM 13g treatments (n=12). Bars represent mean values and error bars represent standard deviation. * $p<0.05$, **** $p<0.0001$ by unpaired t-test (Graphpad Prism 8). (E) **E46K αS neurons treated with 13g LIPE inhibitor have reduced phospholipid-incorporated monounsaturated FAs.** Samples were harvested for FA analysis and analyzed by gas chromatography. Heat maps and statistical analysis were conducted as per materials & methods section. Baseline abundance (Abd) of each FA species is indicated by a green/gray bar on the left of the heat map. Baseline abundance was calculated based on relative amounts of each FA species in the control cells. Red/Blue heatmap coloring is a representation of a given FA species (as per analysis outlined in materials & methods section) FA species are indicated on the right of the heatmap. FA saturated/unsaturated status is indicated by white/black bars to the right of FA species. (F) **LIPEi increases αS T:M ratio**. Quantification of 60+80kDa/14kDa from Fig 2D. **** $p<0.0001$ by unpaired t-test (Graphpad Prism 8). Bars represent mean values and error bars represent standard deviation. n=11. **(G) Expression of LIPE (hosl-1) is decreased by 1 generation of RNAi in *C.elegans* in UA44.** qPCR of hosl-1 (LIPE) as per Materials & Methods section. Bars represent mean values and error bars represent standard error of mean. ** $p<0.01$, unpaired t-test. N=4. **(H) Expression of LIPE (hosl-1) is decreased by 2 generations of RNAi in *C.elegans* in UA44.** ** As (G) but 2 generations of RNAi. * $p<0.05$, unpaired t-test. N=4. **(I) Expression of LIPE (hosl-1) is decreased by 2 generations of RNAi in *C.elegans* in BY250.** As (H) but in BY250. * $p<0.05$, unpaired t-test. N=4.

Supplementary Figure 4: Reducing SCD or LIPE reverses PD relevant phenotypes in patient-derived αS triplication neurons

(A) **SCD inhibition (SCDi) restores pSer129 αS levels in patient-derived αS triplication neurons to that of isogenic control neurons.** Cell lysates were immunoblotted to detect and quantify pSer129 αS, total αS and GAPDH control. Bar charts quantify differences between αS triplication neurons, isogenic corrected neurons and αS triplication neurons treated with SCDi 5b. Bars represent mean values and error bars represent standard deviation. (n in graph order: 9, 8, 8). * $p<0.05$ by one-way Anova (Graphpad Prism 8). (B) **LIPE inhibition (LIPEi) (BAY) restores pSer129 αS levels in patient-derived αS triplication neurons to that of isogenic control neurons.** Cell lysates were immunoblotted to detect and quantify pSer129 αS, total αS and GAPDH control. Bar charts quantify differences between αS triplication neurons, isogenic corrected neurons and αS triplication neurons treated with 1 μM LIPEi BAY. Bars represent mean values and error bars represent standard deviation. n in graph order: 6, 7, 7). * $p<0.05$, ** $p<0.001$ by one-way Anova (Graphpad Prism 8). See also S4I. (C) **SCD inhibition (SCDi) reduces UPR defects of patient-derived αS triplication neurons to that of isogenic control neurons.** Cell lysates were blotted to detect and quantify UPR master regulators, Ire1α and PERK, and GAPDH control. Bar charts quantify differences between αS triplication neurons, isogenic corrected neurons and αS triplication neurons treated with SCDi 5b. Bars represent mean values and error bars represent standard deviation. (n in graph order: 9, 10, 10). * $p<0.05$, ** $p<0.01$, *** $p<0.001$ by one-way Anova (Graphpad Prism 8). (D) **Inhibition of LIPE (BAY) (LIPEi) increases αS T:M ratios of patient-derived αS triplication neurons to that of isogenic control neurons.** Patient-derived αS triplication neurons and isogenic corrected controls were treated with 1 μM BAY LIPE inhibitor (or DMSO control) then subjected to 0.5 mM DSG crosslinking. Cell lysates were immunoblotted to detect and quantify αS14, αS60 and DJ-1 (crosslinking control). (n=6). Multimer/14kDa refers to 60+80/14kDa. Bar charts quantify

differences between patient-derived αS triplication neurons and LIPEi treated αS triplication neurons. Bars represent mean values and error bars represent standard deviation. * $p<0.05$ by t-test (Graphpad Prism 8). See also S4I. (E) **Blot as a visual representation (of graphs presented in Fig. 4C, S7D) of similar changes in αS T:M ratios by inhibition of LIPE using 13g and BAY inhibitors.** Cell lysates were immunoblotted to detect and quantify αS and DJ1. (F) **LIPE inhibition (LIPEi) restores the FA profile of patient αS triplication neurons to that of the isogenic corrected neurons.** Untreated and treated (1 µM BAY) patient-derived αS triplication and corrected neurons were differentiated to DIV24. Samples were harvested for FA analysis and analyzed by gas chromatography. Heat maps and statistical analysis were conducted as per materials & methods section. Baseline abundance (Abd) of each FA species is indicated by a green/gray bar on the left of the heat map. Baseline abundance was calculated based on relative amounts of each FA species in the control cells. Red/Blue heatmap coloring is a representation of a given FA species (as per analysis outlined in materials & methods section). FA species are indicated on the right of the heatmap. FA saturated/unsaturated status is indicated by white/black bars to the right of FA species. (G) **Cell viability is not impacted by 13g LIPE inhibition**. Cell viability of patient-derived αS triplication neurons treated with 5 µM 13g was assayed by LDH release (Promega, G1780) relative to control (DMSO) treated neurons cells. Y-axis is viability: % LDH released relative to the control. Bars represent mean values and error bars represent standard deviation (n=6). (H) **Cell viability is not impacted by BAY LIPE inhibition**. Cell viability of patient-derived αS triplication neurons treated with 1 µM BAY was assayed by LDH release (Promega, G1780) relative to control (DMSO) treated neurons cells. Y-axis is viability: % LDH released relative to the control. Bars represent mean values and error bars represent standard deviation (n=6). (I) **Patient-derived αS triplication neurons treated**

with 5b SCD inhibitor have reduced phospholipid-incorporated monounsaturated FA (C18:1n9). Samples were harvested for FA analysis and analyzed by gas chromatography. Heat maps and statistical analysis were conducted as per materials & methods section. Baseline abundance (Abd) of each FA species is indicated by a green/gray bar on the left of the heat map. Baseline abundance was calculated based on relative amounts of each FA species in the control cells. Red/Blue heatmap coloring is a representation of a given FA species (as per analysis outlined in materials & methods section) FA species are indicated on the right of the heatmap. See S4A and S4C. FA saturated/unsaturated status is indicated by white/black bars to the right of FA species.

Supplementary Figure 5: LIPEi treatment does not alter αS cellular localization in patient-derived αS triplication neurons

(A) **LIPEi treatment does not alter αS cellular localization in αS triplication neurons**. Patient-derived αS triplication neurons (Tripl), isogenic corrected neurons (Corr) and patient-derived neurons treated with LIPEi (BAY, 1μM) (Tripl+LIPEi) were differentiated to D25, fixed in PFA and analyzed by ICC confocal microscopy (see Materials & Methods section). DAPI, Tau, αS and LDs were visualized (using consistent imaging parameters for all images and all samples in all channels for accurate analysis). Images were quantified as per Materials & Methods. Representative images are shown. (B) **αS Cellular Localization is not altered upon LIPEi treatment.** αS images from S5A were over-exposed (min/max display parameters consistently set at 0 and 100 in ImageJ for all images shown) for illustrative purposes. (C) **Quantification of total αS in neurons demonstrates an expected higher αS expression in patient-derived αS triplication neurons relative to corrected neurons**. **LIPEi treatment of**

patient-derived αS triplication neurons does not alter total αS expression. Total αS expression was analyzed from αS microscopy generated for Fig S5A (n in graph order:36,44,43). Bars represent mean values and error bars represent standard deviation. Statistical significant was determined by One-way Anova (Graphpad Prism 8). (D & E) **αS does not re-localize to lipid droplets upon LIPEi**. Co-Localization of LDs with αS (D) and αS with LD (E) was determined using Mander's overlap coefficients (MOC) (see Materials & Methods).

Supplementary Figure 6: LIPEi treatment does not alter αS cellular localization in patient-derived αS triplication neurons

(A) **LIPEi treatment does not alter αS cellular localization in αS triplication neurons**. Images and protocols are per Fig S5A but further magnified. (B) **αS cytosol: membrane distribution is comparable in αS triplication neurons and isogenic corrected neurons.** Sequential extraction was performed per [77]. GAPDH (cytosol) and calnexin (membrane) are used as extraction and normalizing controls. Bar charts quantify ratios of cytosolic:membrane αS and GAPDH:calnexin extraction controls of triplication neurons (n:6) and isogenic corrected neurons (n:12). Bars represent mean values and error bars represent standard deviation. No significant difference by unpaired t-test (Graphpad Prism 8). (C) **Total αS is higher in αS triplication neurons than isogenic corrected neurons**. Cell lysates from (B) were analyzed to quantify combined cytosolic and membrane αS fractions (total αS) and combined extraction controls (total control) were used to normalize data. Bar charts quantify differences between total αS in patient-derived αS triplication neurons (n:6) and isogenic corrected neurons (n:12). Bars represent mean values and error bars represent standard deviation. **** $p<0.0001$ by t-test (Graphpad Prism 8).

Supplementary Figure 7: LIPEi (BAY) reduces FA species 18:1 and 16:1 in phospholipid classes PS, PI, PE, PE-O, PC.

(A) TG are increased in patient-derived αS triplication neurons upon LIPEi treatment. Total triglycerides (TG) (pmol) were measured by mass spectrometry lipid profiling in patient-derived αS triplication neurons untreated (Cntrl) and treated with 1µM BAY. Y-axis is % TG relative to the control. Middle line represents mean values (n in graph order: 5,6). $^{**}p<0.01$ by unpaired t-test (Graphpad Prism 8). (B) **PC, PA, PG, PE containing 18:1 are not altered by treatment with 5µM 13g**. Lipid classes were analyzed by mass spectrometry lipid profiling in patient-derived αS triplication neurons untreated (Cntrl) and treated with 5µM 13g. Data for species containing 18:1 FA were analyzed and reported here. Y-axis is % PC, PA, PG or PE (pmol) containing 18:1 relative to the control. Middle line represents mean values and error bars represent standard deviation. values (n in graph order: 5,6). Statistical analysis: unpaired t-test (Graphpad Prism 8). (C) **PS, PE-O, PE, PI classes containing 18:1 species are decreased upon LIPEi.** Lipid classes containing 18:1 FA species were analyzed by mass spectrometry lipid profiling in patient-derived αS triplication neurons untreated (Cntrl) and treated with 1µM BAY. Data for species containing 18:1 FA were analyzed and reported here. Y-axis is % PS, PE-O, PE, or PI (pmol) containing 18:1 relative to the control. Middle line represents mean values and error bars represent standard deviation. values (n in graph order: 5,6). $^{*}p<0.05$, $^{**}p<0.01$, $^{***}p<0.001$ by unpaired t-test (Graphpad Prism 8). (D) **PS, PE-O, PE, PC, PI classes containing 16:1 species are decreased upon LIPEi.** Lipid classes containing 16:1 FA species were analyzed by mass spectrometry lipid profiling in patient-derived αS triplication neurons untreated (Cntrl) and treated with 1µM BAY. Data for species containing 16:1 FA were analyzed

and reported here . Y-axis is % PS, PE-O, PE, PI, or PC (pmol) containing 16:1 relative to the control. Middle line represents mean values and error bars represent standard deviation values (n in graph order: 5,6). * p<0.05, **p<0.01, *** p<0.001 by unpaired t-test (Graphpad Prism 8).

Supplementary Figure 8: TGs increased in patient-derived αS triplication neurons are enriched for monounsaturated FA upon LIPEi treatment.

(A) **TGs increased in patient-derived αS triplication neurons upon LIPEi treatment (Fig 5A) are enriched for unsaturated 18:1 FA upon 13g LIPEi treatment.** Total triglycerides (TG) (pmol) were measured by mass spectrometry lipid profiling in patient-derived αS triplication neurons untreated (Cntrl) and treated with 5µM 13g. Y-axis is % TG (either 18:1, 16:1 or 18:1+16:1) relative to the control. Middle line represents mean values (n in graph order: 5,6). *p<0.05 by unpaired t-test (Graphpad Prism 8). (B) **TG increased in patient-derived αS triplication neurons upon LIPEi treatment (Fig. S7A) are enriched for unsaturated 18:1 and 16:1 FAs upon BAY LIPEi treatment.** Total triglycerides (TG) (pmol) were measured by mass spectrometry lipid profiling in patient-derived αS triplication neurons untreated (Cntrl) and treated with 1µM BAY. Y-axis is % TG (either 18:1, 16:1 or 18:1+16:1) relative to the control. Middle line represents mean values (n in graph order: 5,6). *p<0.05 by unpaired t-test (Graphpad Prism 8). (C) **TG increased in patient-derived αS triplication neurons upon LIPEi treatment (Fig 5A) are enriched for saturated 16:0 FA upon 13g LIPEi treatment.** Total triglycerides (TG) (pmol) were measured by mass spectrometry lipid profiling in patient-derived αS triplication neurons untreated (Cntrl) and treated with 5µM 13g. Y-axis is % TG (either 18:0, 16:0 or 18:0+16:0) relative to the control. Middle line represents mean values (n in graph order: 5,6). *p<0.05 by unpaired t-test (Graphpad Prism 8). (D) **TG increased in patient-derived αS triplication neurons upon LIPEi treatment (Fig S7A) are enriched for saturated 16:0 FA**

upon BAY LIPEi treatment. Total triglycerides (TG) (pmol) were measured by mass spectrometry lipid profiling in patient-derived αS triplication neurons untreated (Cntrl) and treated with 1μM BAY. Y-axis is % TG (either 18:1, 16:1 or 18:1+16:1) relative to the control. Middle line represents mean values (n in graph order: 5,6). *$p<0.05$ by unpaired t-test (Graphpad Prism 8).

Table 2.0

Fig 1C			
Chain Length	Saturation Status	No. Double Bonds	Fatty Acid Annotation
18	Unsaturated	1	c18_1n9
16	Unsaturated	1	c16_1n9
16	Saturated	0	c16_0
18	Unsaturated	1	c18_1n7
Fig 2A			
Chain Length	Saturation Status	No. Double Bonds	Fatty Acid Annotation
18	Unsaturated	1	c18_1n9
18	Unsaturated	1	c18_1n7
16	Unsaturated	1	c16_1n9
16	Saturated	0	c16_0
Fig 4F			
Chain Length	Saturation Status	No. Double Bonds	Fatty Acid Annotation
18	Unsaturated	1	c18_1n9
16	Unsaturated	1	c16_1n9
18	Unsaturated	1	c18_1n7
18	Saturated	0	c18_0
16	Unsaturated	1	c16_1n7
14	Saturated	0	c14_0
16	Saturated	0	c16_0
20	Unsaturated	1	c20_1n9
Supplemental Fig 1D			
Chain Length	Saturation Status	No. Double Bonds	Fatty Acid Annotation
18	Unsaturated	1	c18_1n9
16	Unsaturated	1	c16_1n9
16	Saturated	0	c16_0
18	Unsaturated	1	c18_1n7
16	Unsaturated	1	c16_1n7
18	Saturated	0	c18_0
14	Saturated	0	c14_0
20	Unsaturated	1	c20_1n9
Supplemental Fig 1G			
Chain Length	Saturation Status	No. Double Bonds	Fatty Acid Annotation
18	Unsaturated	1	c18_1n9
18	Unsaturated	1	c18_1n7
16	Unsaturated	1	c16_1n9
16	Unsaturated	1	c16_1n7
18	Saturated	0	c18_0

14	Saturated	0	c14_0
16	Saturated	0	c16_0
20	Unsaturated	1	c20_1n9
Supplemental Fig 2F			
Chain Length	Saturation Status	No. Double Bonds	Fatty Acid Annotation
18	Unsaturated	1	c18_1n9
18	Unsaturated	1	c18_1n7
16	Unsaturated	1	c16_1n9
16	Saturated	0	c16_0
20	Unsaturated	1	c20_1n9
18	Saturated	0	c18_0
14	Saturated	0	c14_0
16	Unsaturated	1	c16_1n7
Supplemental Fig 2H			
Chain Length	Saturation Status	No. Double Bonds	Fatty Acid Annotation
18	Unsaturated	1	c18_1n9
18	Unsaturated	1	c18_1n7
16	Unsaturated	1	c16_1n9
16	Unsaturated	1	c16_1n7
18	Saturated	0	c18_0
14	Saturated	0	c14_0
16	Saturated	0	c16_0
20	Unsaturated	1	c20_1n9
Supplemental Fig 3A			
Chain Length	Saturation Status	No. Double Bonds	Fatty Acid Annotation
18	Unsaturated	1	c18_1n9
18	Unsaturated	1	c18_1n7
16	Unsaturated	1	c16_1n9
18	Saturated	0	c18_0
14	Saturated	0	c14_0
16	Saturated	0	c16_0
20	Unsaturated	1	c20_1n9
16	Unsaturated	1	c16_1n7
Supplemental Fig 3B			
Chain Length	Saturation Status	No. Double Bonds	Fatty Acid Annotation
18	Unsaturated	1	c18_1n9
18	Unsaturated	1	c18_1n7
16	Unsaturated	1	c16_1n9
16	Saturated	0	c16_0

20	Unsaturated	1	c20_1n9
14	Saturated	0	c14_0
16	Unsaturated	1	c16_1n7
18	Saturated	0	c18_0
Supplemental Fig 3E			
Chain Length	Saturation Status	No. Double Bonds	Fatty Acid Annotation
18	Unsaturated	1	c18_1n9
16	Unsaturated	1	c16_1n9
18	Unsaturated	1	c18_1n7
20	Unsaturated	1	c20_1n9
14	Saturated	0	c14_0
16	Saturated	0	c16_0
18	Saturated	0	c18_0
16	Unsaturated	1	c16_1n7
Supplemental Fig 4F			
Chain Length	Saturation Status	No. Double Bonds	Fatty Acid Annotation
18	Unsaturated	1	c18_1n9
16	Unsaturated	1	c16_1n9
18	Unsaturated	1	c18_1n7
16	Unsaturated	1	c16_1n7
20	Unsaturated	1	c20_1n9
18	Saturated	0	c18_0
16	Saturated	0	c16_0
14	Saturated	0	c14_0
Supplemental Fig 4I			
Chain Length	Saturation Status	No. Double Bonds	Fatty Acid Annotation
18	Unsaturated	1	c18_1n9
18	Unsaturated	1	c18_1n7
16	Unsaturated	1	c16_1n9
16	Unsaturated	1	c16_1n7
18	Saturated	0	c18_0
14	Saturated	0	c14_0
16	Saturated	0	c16_0
20	Unsaturated	1	c20_1n9
